# Ambient aqueous-phase synthesis of covalent organic frameworks for degradation of organic pollutants†
†Electronic supplementary information (ESI) available: Procedure for the preparation of COFs, SEM images, FT-IR spectra, solid-state ^13^C NMR spectra, TGA analysis, stability test, PXRD patterns and structures, unit cell parameters and fractional atomic coordinates, and BET plots. See DOI: 10.1039/c9sc03725j


**DOI:** 10.1039/c9sc03725j

**Published:** 2019-10-16

**Authors:** Yaozu Liu, Yujie Wang, Hui Li, Xinyu Guan, Liangkui Zhu, Ming Xue, Yushan Yan, Valentin Valtchev, Shilun Qiu, Qianrong Fang

**Affiliations:** a State Key Laboratory of Inorganic Synthesis and Preparative Chemistry , Jilin University , Changchun 130012 , China . Email: qrfang@jlu.edu.cn; b Department of Chemical and Biomolecular Engineering , Center for Catalytic Science and Technology , University of Delaware , Newark , DE 19716 , USA; c Normandie Univ , ENSICAEN , UNICAEN , CNRS , Laboratoire Catalyse et Spectrochimie , 6 Marechal Juin , 14050 Caen , France

## Abstract

The development of a mild, low cost and green synthetic route for covalent organic frameworks (COFs) is highly desirable in order to open the door for practical uses of this new family of crystalline porous solids. Herein, we report a general and facile strategy to prepare a series of microporous or mesoporous COFs by a β-ketoenamine based Michael addition–elimination reaction in aqueous systems at ambient temperature and pressure. This synthesis method not only produces highly crystalline and porous COFs, but also can be carried out with a high reaction rate (only 30 min), high yields (as high as 93%) and large-scale preparation (up to 5.0 g). Furthermore, an Fe(ii)-doped COF shows impressive performance in the oxidative degradation of organic pollutants in aqueous medium. This research thus provides a promising pathway to large-scale green preparation of COFs and their potential application in environmental remediation.

## Abstract 2

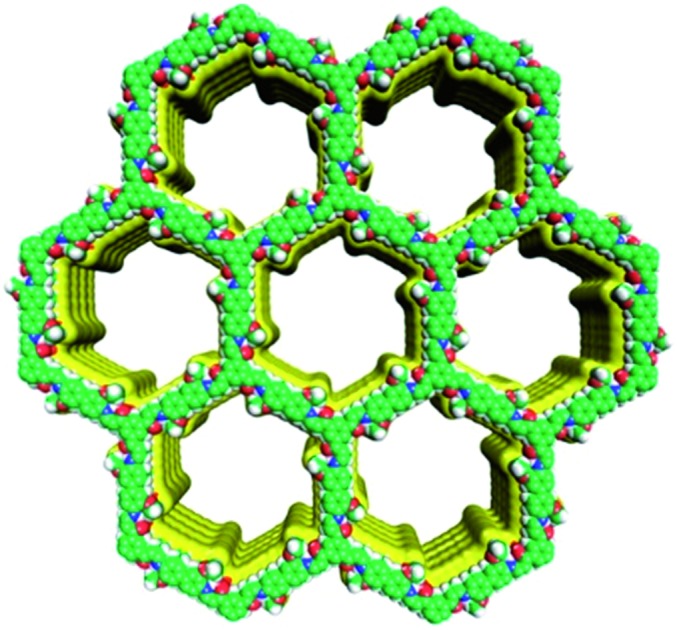
Herein we report the first case of a β-ketoenamine based Michael addition–elimination reaction as well as ambient aqueous-phase synthesis of highly crystalline COF materials.

## Introduction

Covalent organic frameworks (COFs) as a new class of fascinating crystalline porous materials are composed of light elements, such as C, H, N, B and O, and connected by covalent bonds.^1^ Due to their well-defined pore geometry, high surface area, and tunable framework composition, COFs hold great promise in a variety of potential applications, including gas adsorption and separation,^2^ heterogeneous catalysis,^3^ optoelectronic and electrical energy storage devices,^4^ and several others.^5^ Over the past decade, COFs have been obtained by limited reversible chemical reactions, typically the formation of boroxine,1*a* boronate-ester,^6^ imine,^7^ imide,^8^ triazine,^9^ alkene^10^ and dioxin linkage.^11^ Recently, Perepichka and co-workers reported novel 2D π-conjugated COFs as crystalline powders and exfoliated micron-size sheets by using a new dynamic polymerization based on Michael addition–elimination reaction of structurally diverse β-ketoenols with amines.^12^ Furthermore, the classical synthesis of COFs so far is restricted to the solvothermal synthesis, which is implemented in sealed tubes with raising temperatures and pressures, and needs complicated operations and high energy expenditure. Green synthesis using mild reaction conditions and nontoxic precursors, as a reliable, sustainable and eco-friendly protocol, has attracted extensive attention for reducing destructive effects related to traditional methods in the laboratory and industry.^13^ We have exploited a fast, ambient temperature and pressure ionothermal synthesis of COFs;^14^ however, the high cost of ionic liquids hinders the application of this method for large-scale preparation of COFs. Therefore, the development of a simple, low cost and green synthetic strategy for COFs is still highly beneficial.^15^

Taking these considerations in mind, we herein report a general and environmentally benign approach to construct microporous and mesoporous COFs by a β-ketoenamine based Michael addition–elimination reaction in aqueous systems under ambient conditions. On the basis of this method, a series of highly crystalline COFs with pore sizes from 1.59 to 2.92 nm, denoted as JUC-520 (JUC = Jilin University China), JUC-521, JUC-522 and JUC-523, were successfully prepared. Notably, JUC-521 can be obtained in a short period of time (about 30 min) with a high yield (>93%) and by large-scale preparation (up to 5.0 g). Moreover, a metal-doped COF, JUC-521-Fe, shows impressive performance in the degradation of toxic organic pollutants in aqueous solution. To the best of our knowledge, this study is the first case of a β-ketoenamine based Michael addition–elimination reaction as well as ambient aqueous-phase synthesis of highly crystalline COF materials.

## Results and discussion

**Scheme 1:**
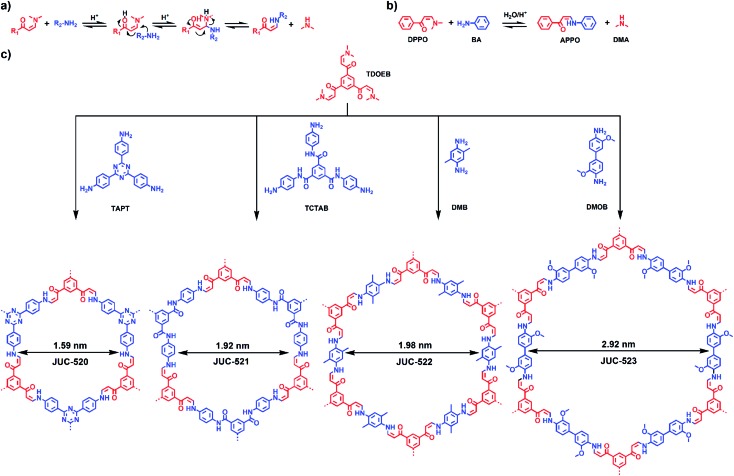
Strategy for ambient aqueous-phase synthesis of COFs. (a) Mechanistic pathway of the Michael addition–elimination reaction of β-ketoenamines and aromatic amines. (b) Model reaction between DPPO and BA to form APPO and DMA. (c) Condensation of TDOEB and TAPT, TCTAB, DMB or DMOB to give JUC-520, JUC-521, JUC-522 or JUC-523 with different pores.

Our strategy for preparing COFs in ambient aqueous systems involves the Michael addition–elimination reaction of β-ketoenamines and aromatic amines. Scheme 1a shows the mechanistic pathway of this reaction. The by-products of this reversible process are organic amines, which can react with acetic acid to form dimethylamine acetate and a small amount of dimethylacetamide (DMAC, Section S1.11, ESI†). Thus, these by-products can accelerate the reaction to generate high quality COF materials. Based on this strategy, the model reaction is implemented between 3-(dimethylamino)-1-phenyl-2-propen-1-one (DPPO) and benzenamine (BA) to form 3-anilino-1-phenyl-2-propen-1-one (APPO) and a by-product, dimethylamine (DMA, Scheme 1b). In pursuit of COFs, 1,3,5-tris(3-dimethylamino-1-oxoprop-2-en-yl)benzene (TDOEB) is chosen as a *C*_3_-symmetric β-ketoenamine-based node. Thus, the condensation of TDOEB and two other *C*_3_-symmetric knots, 1,3,5-tris(4-aminophenyl)triazine (TAPT) and 1,3,5-tricarboxylic acid-tris(4-amino-phenyl-amide)benzene (TCTAB), or two *C*_2_-symmetric linkers, 2,5-dimethyl-1,4-benzenediamine (DMB) and 3,3′-dimethoxybenzidine (DMOB), will give hexagonal microporous JUC-520 (1.59 nm), JUC-521 (1.92 nm) and JUC-522 (1.98 nm) as well as mesoporous JUC-523 (2.92 nm), respectively (Scheme 1c).

Typically, the syntheses were carried out by suspending TDOEB and TAPT, TCTAB, DMB or DMOB in aqueous solution with acetic acid as the catalyst, followed by keeping at ambient temperature and pressure to produce crystalline solids. All COFs can be synthesized within 8 h, which is substantially shorter than those from the traditional solvothermal method (3–7 days). To further explore the aqueous-phase synthesis of COFs, JUC-521 was selected as an example to study the influence of different reaction conditions including temperature, concentration of catalysts, and reaction time (Fig. S1–S3, ESI†). Remarkably, highly crystalline JUC-521 can be obtained at room temperature in 6 M acetic acid aqueous solution within only 30 min, accompanied by a high yield (93%). Moreover, scale-up synthesis of JUC-521 (∼5.0 g) can be successfully performed by increasing the amount of reactants and solvents (Fig. S4, ESI†). Thus, this simple, low cost and scale-up green process suggests that mass production of COFs can be achieved based on this reaction strategy.

The structural features of the new COFs were determined by complementary methods. The crystal morphology was observed by scanning electron microscopy (SEM). Relatively small building complex aggregates (*ca.* 0.2 μm) are characteristic of all samples (Fig. S5–S8, ESI†). Fourier transform infrared (FT-IR) spectra displayed the disappearance of N–H stretching of the primary amines (∼3450 cm^–1^) and N–CH_3_ stretching of TDOEB (1438 cm^–1^), which confirms that the reaction has taken place (Fig. S9–S13, ESI†). The solid state ^13^C cross-polarization magic-angle-spinning (CP/MAS) NMR spectra further demonstrated that all COFs showed a characteristic signal at ∼145 ppm, corresponding to the α-carbon of enamine (Fig. S14–S17, ESI†). According to the thermogravimetric analysis (TGA, Fig. S18–S21, ESI†), these COFs are thermally stable up to 350 °C. Their crystalline structures can be preserved in a variety of organic solvents as well as acidic (1.0 M HCl) and basic (1.0 M NaOH) aqueous solutions for 3 days (Fig. S22–S25, ESI†). In addition, a partial loss of the PXRD signal intensity was also observed after soaking in acidic and basic aqueous solutions, which could be attributed to structural rearrangement induced by partial hydrolysis.

**Fig. 1:**
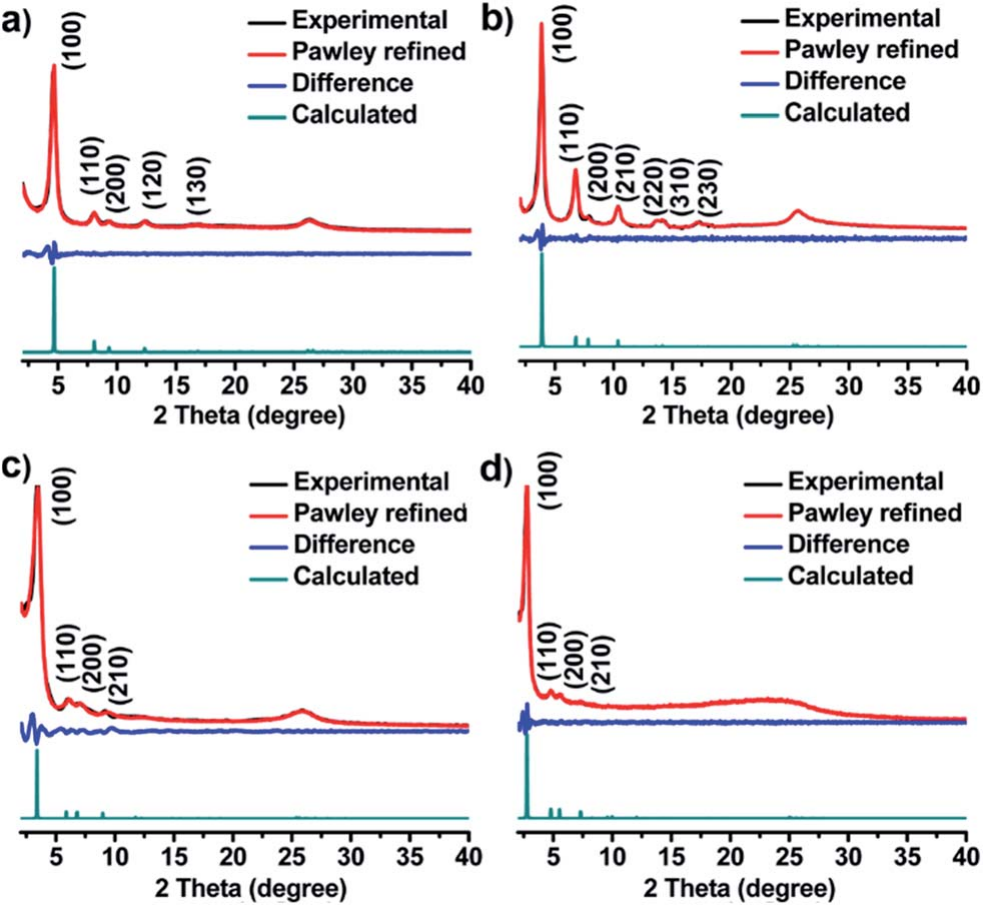
PXRD patterns of JUC-520 (a), JUC-521 (b), JUC-522 (c) and JUC-523 (d).

**Fig. 2:**
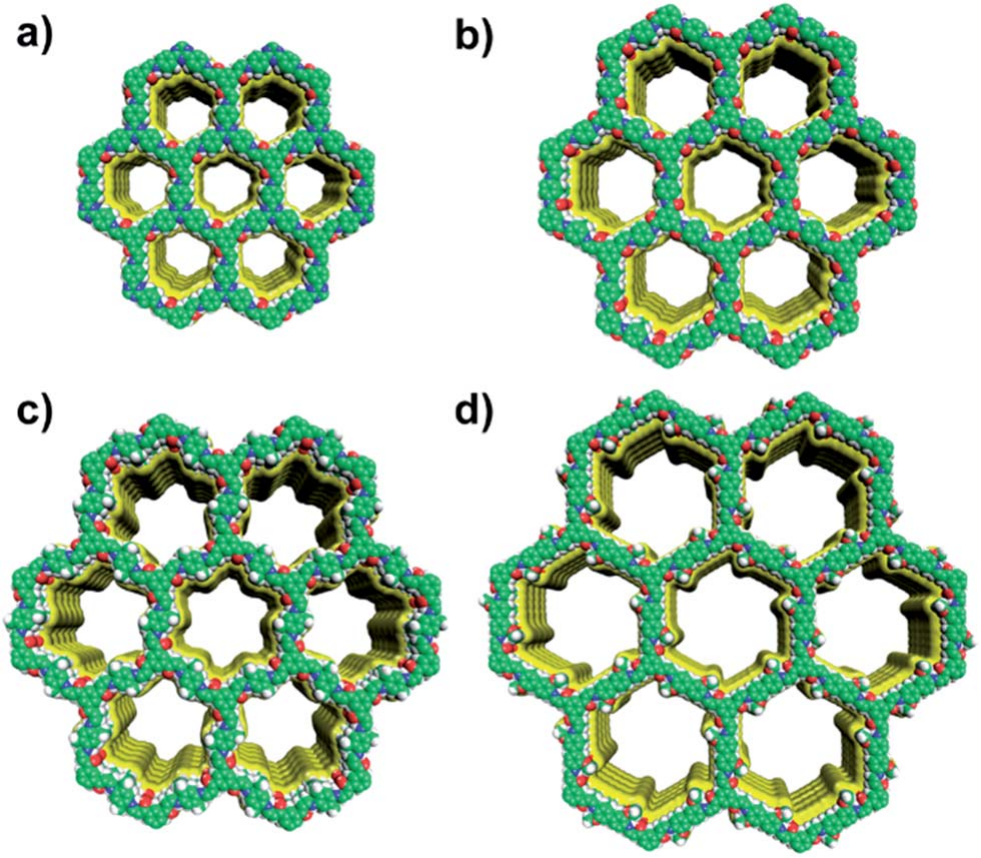
Structural representations of JUC-520 (a), JUC-521 (b), JUC-522 (c), and JUC-523 (d). C, green; H, gray; N, blue; O, red.

The unit cell parameters of COFs were resolved by using the powder X-ray diffraction (PXRD) patterns combined with structural simulations. After a geometrical energy minimization by using the Materials Studio software package,^16^ their unit cell parameters based on the AA stacking boron nitride net (**bnn**) were obtained (*a* = *b* = 22.4795 Å, *c* = 3.5114 Å, *α* = *β* = 90° and *γ* = 120° for JUC-520; *a* = *b* = 26.4476 Å, *c* = 3.5064 Å, *α* = *β* = 90° and *γ* = 120° for JUC-521; *a* = *b* = 30.0949 Å, *c* = 3.5075 Å, *α* = *β* = 90° and *γ* = 120° for JUC-522; and *a* = *b* = 36.9035 Å, *c* = 3.5605 Å, *α* = *β* = 90° and *γ* = 120° for JUC-523, respectively). The experimental PXRD peaks were in good agreement with the simulated ones (Fig. 1). Full profile pattern matching (Pawley) refinements were also applied. Peaks at 4.66, 8.07, 9.31, 12.38 and 16.64° 2*θ* for JUC-520 correspond to the (100), (110), (200), (120) and (130) Bragg peaks of the space group *P*6 (no. 174); peaks at 3.87, 6.76, 7.89, 10.36, 13.56, 14.19 and 17.21° 2*θ* for JUC-521 correspond to the (100), (110), (200), (210), (220), (310) and (230) Bragg peaks of the space group *P*6 (no. 174); peaks at 3.41, 6.07, 7.05 and 9.21° 2*θ* for JUC-522 correspond to the (100), (110), (200) and (210) Bragg peaks of the space group *P*6 (no. 168); and peaks at 2.77, 4.79, 5.57 and 7.31° 2*θ* for JUC-523 correspond to the (100), (110), (200) and (210) Bragg peaks of the space group *P*6 (no. 168). The refinement results show unit cell parameters very close to the predictions with ideal agreement factors (*ωR*_p_ = 3.63% and *R*_p_ = 2.52% for JUC-520; *ωR*_p_ = 5.34% and *R*_p_ = 3.92% for JUC-521; *ωR*_p_ = 3.77% and *R*_p_ = 2.45% for JUC-522; and *ωR*_p_ = 3.91% and *R*_p_ = 2.55% for JUC-523). In contrast, simulated PXRD data of alternative staggered 2D arrangements (AB stacking) showed significant deviations compared with the experimental ones (Fig. S26–S37 and Tables S1–S8, ESI†). On the basis of these results, the obtained COFs were proposed to have the expected frameworks with hexagonal micropores for JUC-520, JUC-521 and JUC-522 as well as mesopores for JUC-523 (Fig. 2).

**Fig. 3:**
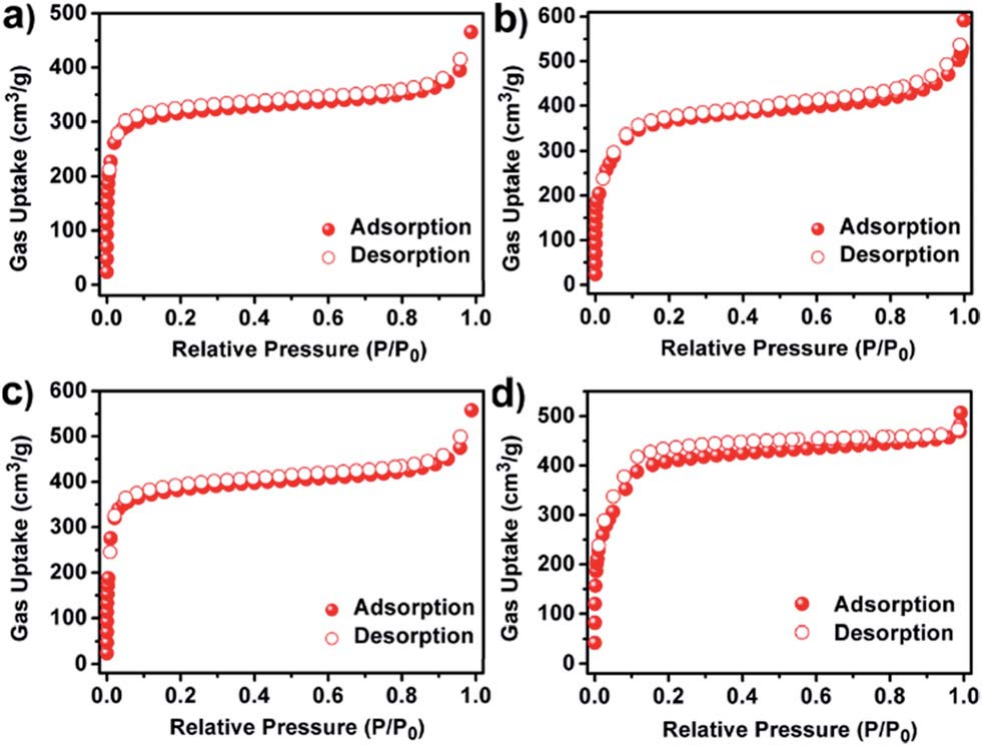
N_2_ adsorption–desorption isotherms of JUC-520 (a), JUC-521 (b), JUC-522 (c), and JUC-523 (d).

The porosity and textural characteristics of the synthesized series of COFs were determined by using nitrogen (N_2_) adsorption–desorption isotherms at 77 K. As shown in Fig. 3a–c, a sharp gas uptake can be observed at low pressure (below 0.05*P*/*P*_0_) for JUC-520, JUC-521 and JUC-522, which reveals their microporous nature. JUC-523 showed a rapid uptake at a low pressure of *P*/*P*_0_ < 0.05, followed by a second step between *P*/*P*_0_ = 0.1 and 0.2 (Fig. 3d), which is characteristic of mesoporous materials. The Brunauer–Emmett–Teller (BET) surface areas of these COFs are 976 m^2^ g^–1^ for JUC-520, 1127 m^2^ g^–1^ for JUC-521, 1182 m^2^ g^–1^ for JUC-522, and 1435 m^2^ g^–1^ for JUC-523 (Fig. S38–S41, ESI†). These values are much higher than those of previous COFs obtained *via* Michael addition–elimination of β-ketoenols with amines (∼500 m^2^ g^–1^),^12^ which can be attributed to their high crystallinity. The total pore volume was calculated at *P*/*P*_0_ = 0.9 to be 0.56 cm^3^ g^–1^ for JUC-520, 0.67 cm^3^ g^–1^ for JUC-521, 0.69 cm^3^ g^–1^ for JUC-522, and 0.88 cm^3^ g^–1^ for JUC-523. Their pore-size distributions were estimated by using nonlocal density functional theory (NLDFT), and showed narrow pore widths (1.53 nm for JUC-520, 1.87 nm for JUC-521, 1.95 nm for JUC-522, and 2.88 nm for JUC-523, Fig. S42–S45, ESI†), which are in good agreement with those from their crystal structures.

Encouraged by the high surface area and abundant β-ketoenamine units, Fe(ii)-loaded JUC-521 (JUC-521-Fe) was subsequently acquired by immersing the pristine material in an ethanol solution of FeSO_4_. The ferrous complexation of JUC-521 could be easily observed by the color change (Fig. S46, ESI†). The energy dispersive X-ray spectroscopy (EDS) mapping image showed the uniform distribution of Fe species in the crystals (Fig. S47, ESI†). FT-IR spectroscopy revealed the strong chelation interaction between β-ketoenamine units and Fe(ii) ions based on the shift of the IR bands for the carbonyl groups from 1662 to 1653 cm^–1^ and amine bonds of enamine from 1221 to 1212 cm^–1^ (Fig. S48, ESI†). The ICP analysis showed that most of the β-ketoenamine units were coordinated to metal ions (see Section S1, ESI†). The porosity of JUC-521-Fe was also evaluated by N_2_ adsorption analysis, which pointed out that the adsorption capacity and pore size were slightly less than those of the parent material (Fig. S49 and S50, ESI†). In addition, the PXRD pattern of JUC-521-Fe indicated that the pristine framework was preserved after the metal incorporation (Fig. S51, ESI†).

**Fig. 4:**
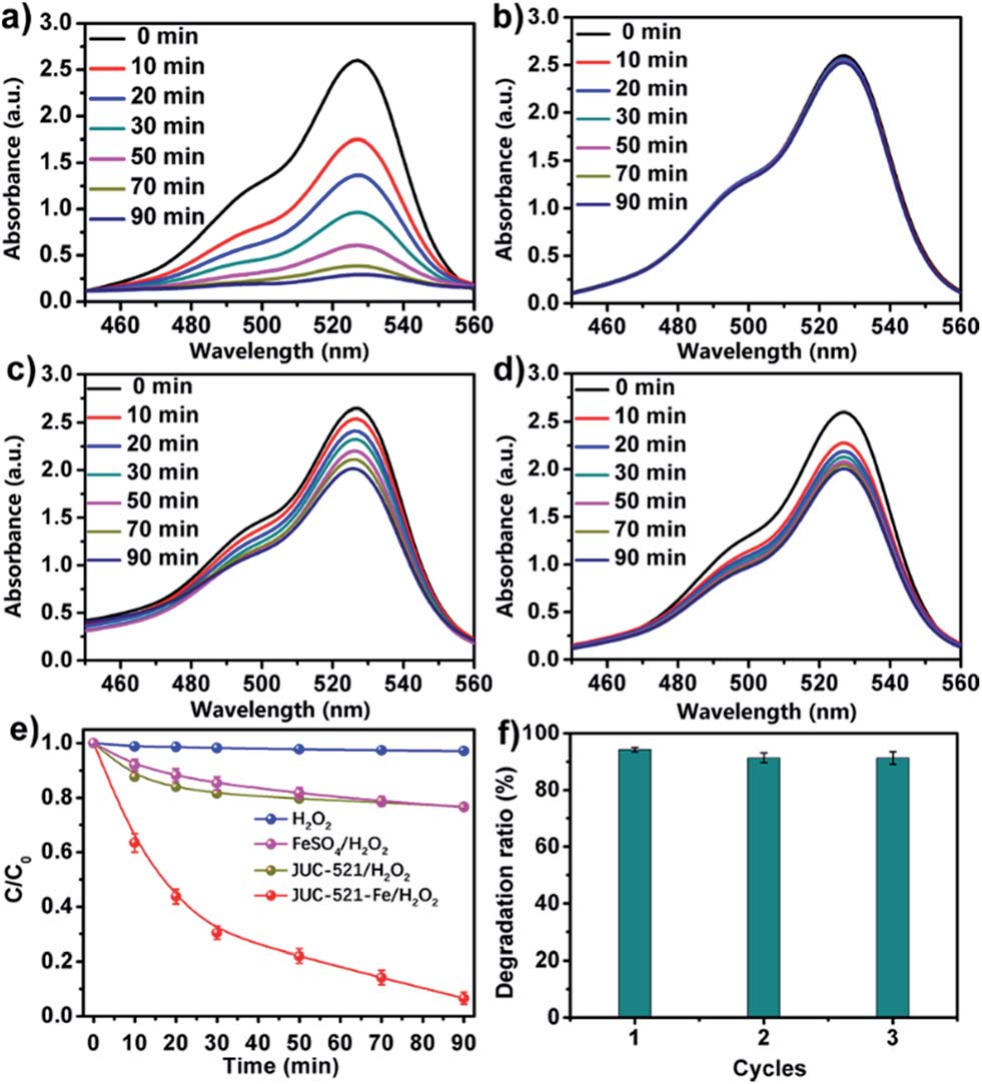
UV-vis spectra of Rh6G aqueous solution in the presence of JUC-521-Fe/H_2_O_2_ (a), H_2_O_2_ (b), FeSO_4_/H_2_O_2_ (c) and JUC-521/H_2_O_2_ (d). (e) Comparison of the degradation abilities of Rh6G for different materials. (f) Recyclability study of JUC-521-Fe.

We further explored the application of JUC-521-Fe in the degradation of organic pollutants in aqueous medium based on the Fenton reaction. It is well known that the Fenton reaction, one of the most popular advanced oxidation processes (AOP), involves an *in situ* generation of hydroxyl or peroxyl radicals (˙OH or HO_2_˙) from hydrogen peroxide (H_2_O_2_) activated by ferrous ions, to degrade hazardous organic compounds.^17^ However, the traditional Fenton reaction was carried out with homogeneous catalysts, which is strongly limited because of poor recyclability and high energy consumption. Herein, we used JUC-521-Fe as a heterogeneous Fenton catalyst for effective degradation of organic pollutants due to its high surface area, chemical stability and abundant catalytic sites. In addition, due to the large channels in JUC-521-Fe, we expect to use this material for adsorption and degradation of large toxic dyes, such as rhodamine 6G (Rh6G), a famous toxic dye as a model substrate. As shown in Fig. 4a, JUC-521-Fe showed a high catalytic performance in the degradation of Rh6G with a reaction rate constant of 2.8 × 10^–2^ min^–1^ and a degradation efficiency of 95% in 90 min, which can be compared with those of the state-of-the-art TiO_2_-based composites, such as TiO_2_ (ref. 18) with a reaction rate constant of 8.9 × 10^–3^ min^–1^ and a degradation efficiency of 63% in 180 min as well as nanosize TiO_2_ (ref. 19) with a reaction rate constant of 1.3 × 10^–2^ min^–1^ and a degradation efficiency of 98% in 180 min, and those of iron-based materials using different porous supports, such as Fe_2_O_3_/active carbon fibers^20^ with a reaction rate constant of 1.0 × 10^–2^ min^–1^ and a degradation efficiency of 90% in 120 min as well as SiO_2_/Fe_3_O_4_ shelled hollow spheres^21^ with a reaction rate constant of 2.6 × 10^–2^ min^–1^ and a degradation efficiency of 95% in 120 min. Furthermore, H_2_O_2_, metal-free pristine material (JUC-521/H_2_O_2_) and FeSO_4_/H_2_O_2_ were also tested under the same conditions. Much lower degradation abilities for Rh6G were observed (Fig. 4b–e), such as only 4% for H_2_O_2_, 19% for JUC-521/H_2_O_2_ and 21% for FeSO_4_/H_2_O_2_ at the same time. The SEM image and PXRD pattern confirmed the retained crystallinity of JUC-521-Fe after the catalytic reaction (Fig. S52 and S53, ESI†). Furthermore, ICP analysis revealed that almost no ferrous ions leached during the degradation (see Section 1, ESI†). As a heterogeneous catalyst, JUC-521-Fe can be isolated by centrifugation and reused at least three times almost without loss of activity (Fig. 4f).

## Conclusions

In conclusion, we have achieved a mild, low cost and green process to prepare a number of microporous or mesoporous COFs by a β-ketoenamine based Michael addition–elimination reaction under ambient synthesis conditions employing aqueous systems. This strategy not only yields highly crystalline and porous COFs, but also offers the advantage of a high reaction rate (30 min), impressive yield (93%) and scalability potential (5.0 g). Moreover, the metal-doped COF, JUC-521-Fe, shows a high reaction rate constant of 2.8 × 10^–2^ min^–1^, a degradation efficiency of 95% in 90 min, and reusability in the degradation of toxic organic pollutants based on Fenton's reaction. This research thus provides a promising synthetic strategy for large-scale production of COFs and expands their potential application in environmental remediation.

## Conflicts of interest

There are no conflicts to declare.

## Supplementary Material

Supplementary information
