# Supplementary material for: Ambient aqueous-phase synthesis of covalent organic frameworks for degradation of organic pollutants
Source: Chem Sci. 2019 Oct 16;10(46):10815–20. doi: 10.1039/c9sc03725j (PMC7066675; doi:10.1039/c9sc03725j)
Supplement: Supplementary file 1 [file SC-010-C9SC03725J-s001.pdf]

Electronic Supplementary Material (ESI) for Chemical Science.
This journal is © The Royal Society of Chemistry 2019

Supplementary information

Ambient Aqueous-Phase Synthesis of Covalent Organic Frameworks for Degradation of Organic Pollutants

Yaozu Liu,[1] Yujie Wang,[1] Hui Li,[1] Xinyu Guan,[1] Liangkui Zhu,[1] Ming Xue,[1] Yushan Yan,[2] Valentin Valtchev,[1,3] Shilun Qiu[1] and Qianrong Fang[1*]

[1] State Key Laboratory of Inorganic Synthesis and Preparative Chemistry, Jilin University, Changchun 130012, China

E-mail: qrfang@jlu.edu.cn

[2] Department of Chemical and Biomolecular Engineering, Center for Catalytic Science and Technology, University of Delaware, Newark, DE 19716, USA

[3] Normandie Univ, ENSICAEN, UNICAEN, CNRS, Laboratoire Catalyse et Spectrochimie, 6 Marechal Juin, 14050 Caen, France

Table of contents

**Section 1**	Methods	S3-10
**Section 2**	Synthesis study	S11-12
**Section 3**	SEM images	S13-14
**Section 4**	FTIR spectra	S15-16
**Section 5**	Solid-state ^{13}C NMR spectra	S17-18
**Section 6**	TGA analysis	S19-20
**Section 7**	Stability test	S21-22
**Section 8**	PXRD patterns and structures	S23-26
**Section 9**	Nitrogen adsorption	S27-29
**Section 10**	Characterization of JUC-521-Fe	S30-32
**Section 11**	Unit cell parameters and fractional atomic coordinates	S33-44
**Section 12**	References	S45

Section 1: Methods

1.1 Materials and characterization. All starting materials and solvents, unless otherwise noted, were obtained from J&K scientific LTD. All the purchased reagents were of 95% and used without further purification. All products were isolated and handled under nitrogen using either glovebox or Schlenk line techniques. ^{1}H NMR spectra were recorded on an AV400 NMR spectrometer. ^{13}C CP/MAS NMR spectra were recorded on an AVIII 500 MHz solid-state NMR spectrometer. The FTIR spectra (KBr) were obtained using a SHIMADZU IRAffinity-1 Fourier transform infrared spectrophotometer. A SHIMADZU UV-2450 spectrophotometer was used for all absorbance measurements. TGA was carried out under nitrogen on a SHIMADZU DTG-60 thermal analyzer at a heating rate of 10 °C min^{-1} to 600 °C with N_2 flow rate of 30 mL min^{-1}. Element analysis was carried out on a Germany Elementar large sample volume element analyzer, vario MACRO cube CHNS. PXRD data were collected on a PANalytical B.V. Empyrean powder diffractometer using a Cu Kα source (λ = 1.5418 Å) over the range of 2θ = 2.0−40.0° with a step size of 0.02° and 2 s per step. The sorption isotherm for N_2 was measured by using a Quantachrome Autosorb-IQ analyzer with ultra-high-purity gas (99.999% purity). Before gas adsorption measurements, each COF (~50.0 mg) was immersed in ethanol for 24 h and then acetone for another 24 h, during which ethanol and acetone were decanted and freshly replenished 3 times, respectively. The acetone was then extracted under vacuum at 100 °C to afford the samples for sorption analysis. To estimate pore size distributions, nonlocal density functional theory (NLDFT) was applied to analyze the N_2 isotherm on the basis of the model of N_2 @77 K on carbon with slit pores and the method of non-negative regularization. The SEM images were obtained on JEOL JSM6700 scanning electron microscope. The TEM images and EDS spectra were obtained on JEM-2100 transmission electron microscopy.

The Fe contents of COF samples were determined by ICP analysis with an IRIS advantage instrument.

1.2 Synthesis of 1,3,5-tris(3-dimethylamino-1-oxoprop-2-en-yl)benzene (TDOEB)[1]

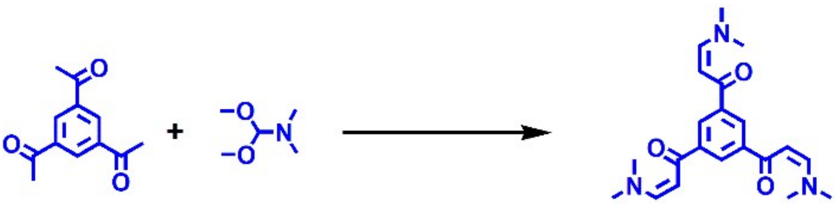

1,3,5-Triacetyl benzene (0.82 g, 4.0 mmol) and *N*,*N*-dimethylformamide diethyl acetal (2.4 g, 12.0 mmol) were dissolved in 10.0 mL DMF and stirred at 90 °C under dry N_2 for 12 hrs. The products were obtained by addition of Et_2O as yellow microcrystals in high yields. The crystals were washed twice with pentane (50.0 mL) and dried in vacuum; yield: 91%; mp 250 °C. Anal. Cald $C_{21}H_{27}N_3O_3$ (369.5): C 68.27, H 7.37, N 11.37; Found C 67.92, H 7.33, N 11.06. ^1H NMR (400 MHz, $CDCl_3$) δ: 2.86, 3.07 [2 s, 18 H, $N(CH_3)_2$], 5.78 (d, 3 H, J = 12.7, COCH=), 7.75 [d, 3 H, =$CHN(CH_3)_2$], 8.46 (s, 3 H, H-2, 4, 6). ^{13}C NMR (100 MHz, $CDCl_3$) δ: 37.3, 45.0 [$N(CH_3)_2$], 92.2 (COCH=), 128.7 (C-2, 4, 6), 140.2 (C-1, 3, 5), 154.4 [=$CHN(CH_3)_2$], 187.6 (C=O).

1.3 Synthesis of 1,3-5-tricarboxylic acid-tris(4-amino-phenyl-amide) benzene (TCTAB)[2]

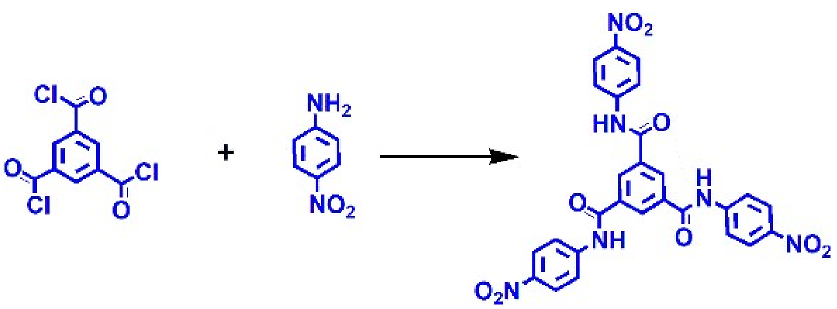

4-Nitroaniline (0.80 g, 5.79 mmol) and 1,3,5-benzenetricarbonyl trichloride (0.47 g, 1.75 mmol) was dissolved in MeCN and stirred at 85 °C. The product was isolated as an off white solid (0.84 g,

84% yield). m.p. decomposed above 300°C; ^{1}H-NMR (400 MHz, DMSO-d_6) 11.18 (s, 1 H, NH), 8.81 (s, 1 H, CH), 8.31 (d, 2 H, CH, J = 9.04 Hz), 8.12 (d, 2H, CH, J = 9.52 Hz).

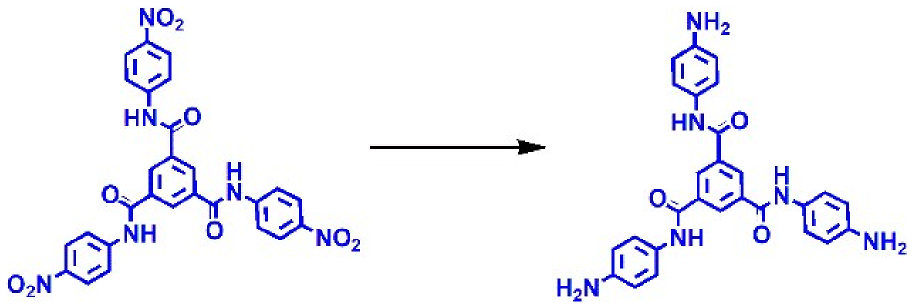

Tris-nitro compound (0.20 g, 0.35 mmol), and hydrazine monohydrate (0.46 g, 9.28 mmol) was suspended in DMF at 95 °C. The product was isolated as a yellow solid (0.13 g, 78% yield). m.p. 202-206°C; ^{1}H-NMR (400 MHz, DMSO-d_6) 10.17 (s, 1H, NH), 8.57 (s, 1H, CH), 7.43 (d, 2H, CH, J = 8.76 Hz), 6.57 (d, 2H, CH, J = 8.80 Hz), 5.04 (s, 2H, NH_2).

1.4 Synthesis of the model compound, 3-anilino-1-phenyl-2-propen-1-one (APPO)

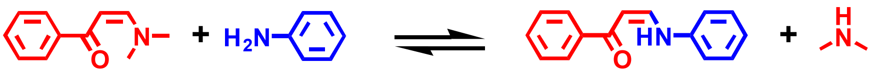

Mixture of DPPO (100.0 mg, 0.57 mmol) and aniline (120.0 mg, 1.29 mmol) were suspended in 6 M aq. acetic acid (50.0 mL) and stirred at ambient temperature and pressure for 30 mins. The product was extracted and recrystallized from a mixture of dichloromethane and n-hexane. Yield 91%; m.p. 138-139°C; ^{1}H-NMR ($CDCl_3$, 400 MHz) 12.13 (d, 1 H, J = 12.28, NH), 7.93 (dd, 2 H, J = 7.95 and 1.86, arom. H), 7.56-7.02 (m, 9 H, arom. H and =CH-N), 6.02 (d, 1 H, J = 7.92, =CHCO).

1.5 Synthesis of JUC-520

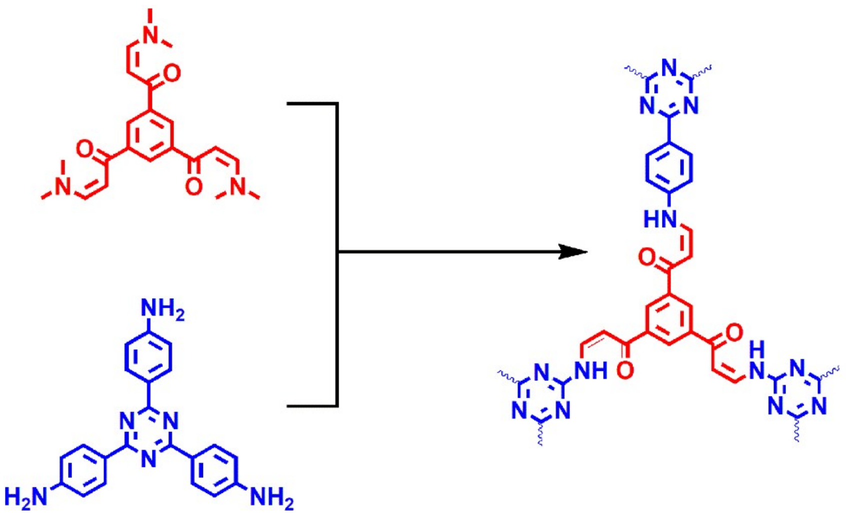

In a plastic centrifuge tube with the volume of 5.0 ml, TDOEB (18.5 mg, 0.05 mmol) and TAPT (17.7 mg, 0.05 mmol) were suspended in 4.0 mL aqueous solution with 0.4 mL acetic acid as the catalyst. The mixture was kept at ambient temperature and pressure for 8 hrs. Then the precipitate was filtered, washed with DMF (3 × 10.0 mL), acetone (3 × 10.0 mL) and n-hexane (3 × 10.0 mL), and dried at 100 °C under vacuum overnight to afford yellow solids (yield 87%). Anal. Cald: C: 73.47; H: 4.08; N: 14.29. Found: C: 73.75; H: 3.96; N: 14.31.

1.6 Synthesis of JUC-521

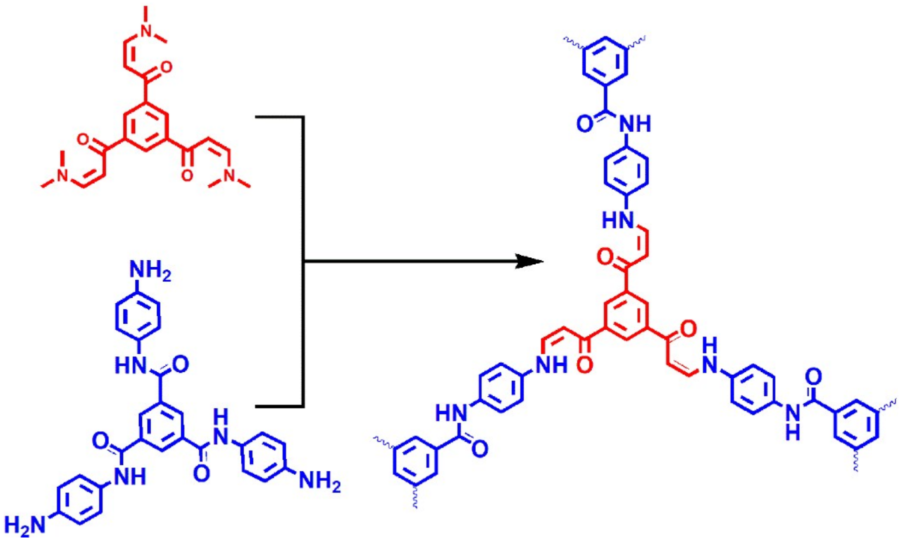

Similar to the synthesis of JUC-520, TDOEB (18.5 mg, 0.05 mmol) and TCTAB (24.0 mg, 0.05

mmol) were suspended in 4.0 mL aqueous solution with 0.4 mL acetic acid as the catalyst in a plastic centrifuge tube with the volume of 5.0 ml. The mixture was kept at ambient temperature and pressure for 8 hrs. Then the precipitate was filtered, washed with DMF (3 × 10.0 mL), acetone (3 × 10.0 mL) and n-hexane (3 × 10.0 mL), and dried at 100 °C under vacuum overnight to afford yellow solids (yield 93%). Anal. Cald: C: 70.59; H: 4.20; N: 11.76. Found: C: 70.37; H: 4.21; N: 11.54. JUC-521 was selected as an example to study the influence of different reaction conditions including temperature (RT, 40, 60 or 80 ºC), concentration of catalyst (1.0, 3.0, 6.0 or 9.0 M HAc), and reaction time (10, 20, 30 or 60 mins). Scale-up synthesis of JUC-521 (~ 5.0 gram) was also carried out by enlarging the amount of reactants and solvents: TDOEB (2.96 g, 8.0 mmol) and TCTAB (2.83 g, 8.0 mmol) in 150.0 mL 6.0 M HAc aqueous solution.

1.7 Synthesis of JUC-522

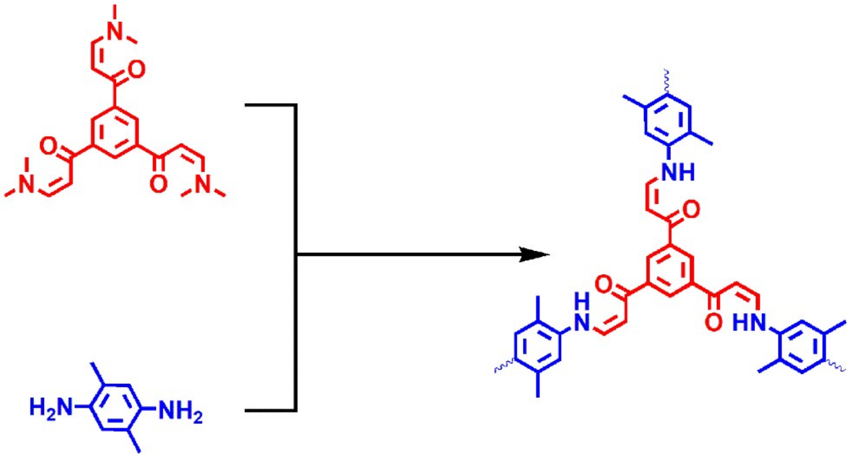

Similar to the synthesis of JUC-520, TDOEB (18.5 mg, 0.05 mmol) and DMB (6.8 mg, 0.05 mmol) were suspended in 4.0 mL aqueous solution with 0.4 mL acetic acid as the catalyst in a plastic centrifuge tube with the volume of 5.0 ml. The mixture was kept at ambient temperature and pressure for 8 hrs. Then the precipitate was filtered, washed with DMF (3 × 10.0 mL), acetone (3 × 10.0 mL) and n-hexane (3 × 10.0 mL), and dried at 100 °C under vacuum overnight to afford red

solids (yield 83%). Anal. Cald: C: 73.97; H: 5.48; N: 9.59. Found: C: 73.75; H: 5.56; N: 9.31.

1.8 Synthesis of JUC-523

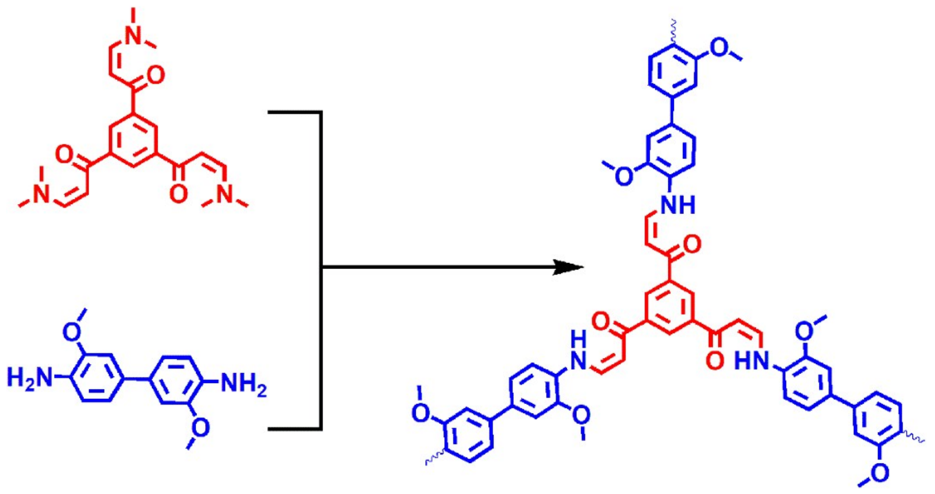

Similar to the synthesis of JUC-520, TDOEB (18.5 mg, 0.05 mmol) and DMOB (12.2 mg, 0.05 mmol) were suspended in 4.0 mL aqueous solution with 0.4 mL acetic acid as the catalyst in a plastic centrifuge tube with the volume of 5.0 ml. The mixture was kept at ambient temperature and pressure for 8 hrs. Then the precipitate was filtered, washed with DMF (3 × 10.0 mL), acetone (3 × 10.0 mL) and n-hexane (3 × 10.0 mL), and dried at 100 °C under vacuum overnight to afford red solids (yield 81%). Anal. Cald: C: 71.99; H: 5.03; N: 7.00. Found: C: 70.98; H: 4.56; N: 7.03.

1.9 Synthesis of JUC-521-Fe[3]

JUC-521-Fe was prepared by immersing 100.0 mg JUC-521 with 200.0 mg iron(II) sulfate heptahydrate in 20.0 mL mixed solution (H_2O: ethanol = 1:1) for 24 hrs. The powder was filtered and washed with H_2O for 3 times and dried at 100 °C under vacuum overnight to afford JUC-521-Fe as a brown solid. The Fe content in JUC-521-Fe was 12.32 wt% as determined by ICP, which means the molar ratio of Fe to enaminone are about 91%. Anal. Cald for

$[(C_{14}N_2O_2Fe_{0.91}H_{10})(SO_4)_{0.91}]_n$: C: 44.64; H: 2.66; N: 7.44; Fe: 13.54. Found: C: 43.75; H: 2.56; N: 7.31; Fe: 12.32. Found (after the degradation experiment): C: 44.21; H: 2.63; N: 7.49; Fe: 11.75.

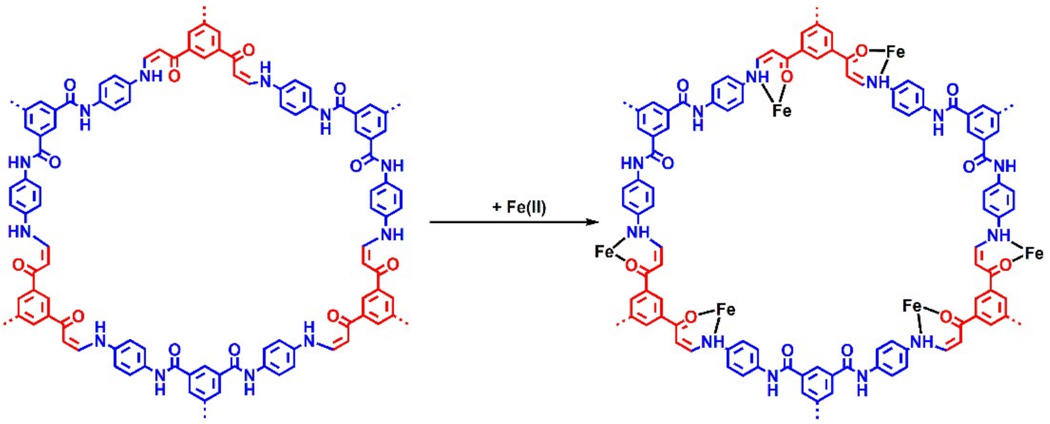

1.10 JUC-521-Fe as a heterogeneous Fenton catalyst

Rh6G degradation experiments were performed in a glass tube (20.0 mL) containing 10.0 mL solution at initial pH = 7 with Rh6G (C_0 = 17.5 mg/L), 15% H_2O_2, and 10.0 mg JUC-521-Fe. The experiments were carried out under standard conditions (298 K, 1 atm and no control of light intensity). As a comparison, 15% H_2O_2, metal-free pristine material (10.0 mg JUC-521 in 15% H_2O_2) and 3.4 mg $FeSO_4{\bullet}7H_2O$ (the same amount of Fe with JUC-521-Fe) in 15% H_2O_2 were tested under the same conditions, respectively.

1.11 Analysis for by-product[4]

The synthesis is the same as above for JUC-520, but the solvent has been changed to D_2O. The supernatant was taken for NMR test. A small amount of dimethylacetamide (DMAC) was found based on the reaction of acetic acid and dimethylamine. ^{1}H NMR for DMAC (D_2O, 400 MHz): 2.12 (s, 3H CH_3CO), 3.06 (s, 3H NCH_3), 2.90 (s, 3H NCH_3).

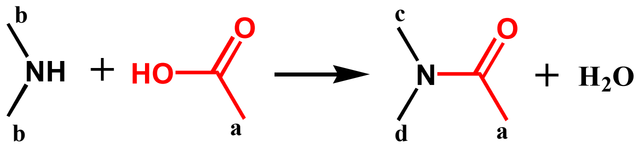

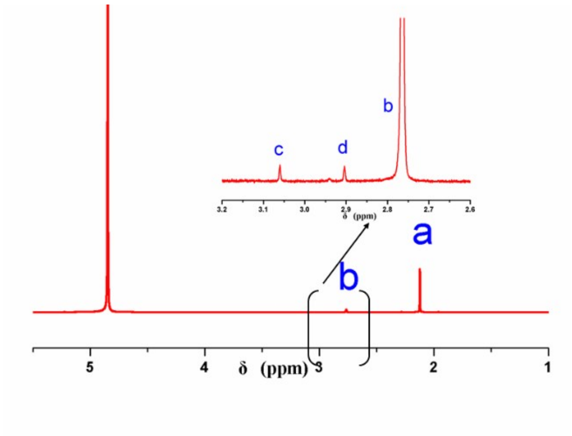

^{1}H NMR for DMAC

Section 2: Synthesis study

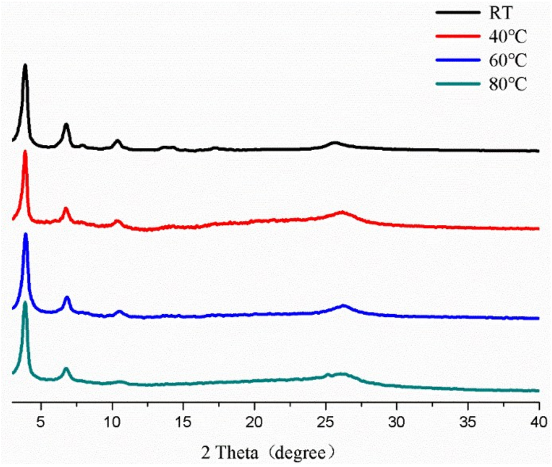

Figure S1. PXRD patterns of JUC-521 based on different reaction temperature (RT, 40, 60 or 80 ºC) in 6.0 M HAc aqueous solution for 30 mins.

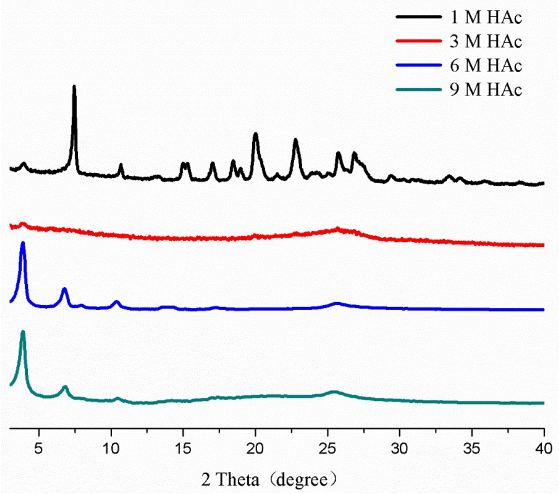

Figure S2. PXRD patterns of JUC-521 based on different concentration of catalyst (1.0, 3.0, 6.0 or 9.0 M HAc) at RT for 30 mins.

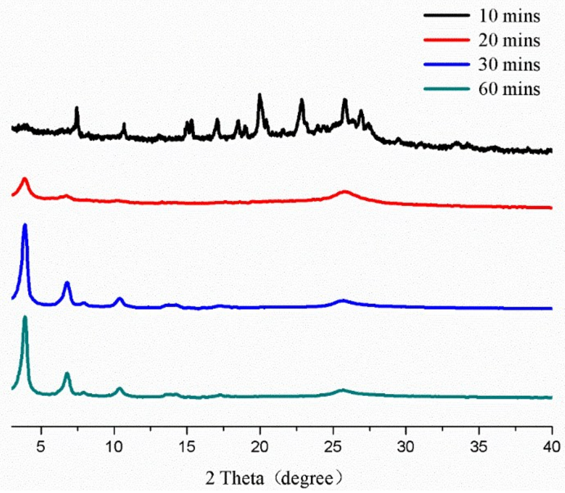

Figure S3. PXRD patterns of JUC-521 based on different reaction time (10, 20, 30 or 60 mins) in 6.0 M HAc aqueous solution at RT.

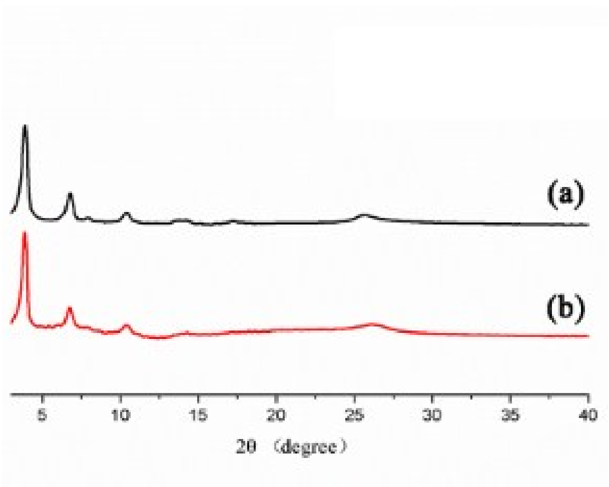

Figure S4. PXRD patterns of JUC-521 based on normal (~0.03 g, a) and scale-up (~5.0 g, b) synthesis by enlarging the amount of reactants and solvents.

Section 3: SEM images

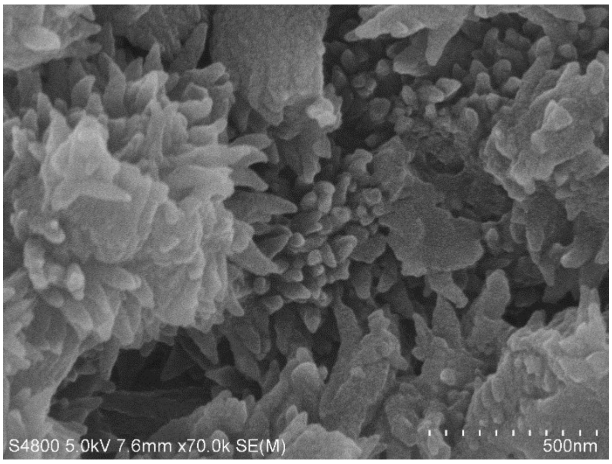

Figure S5. SEM image of JUC-520.

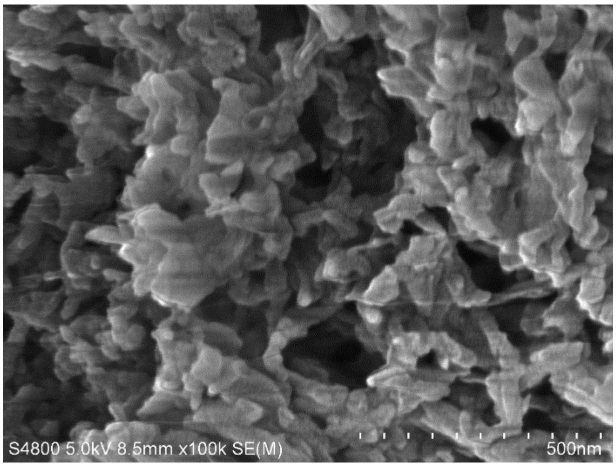

Figure S6. SEM image of JUC-521.

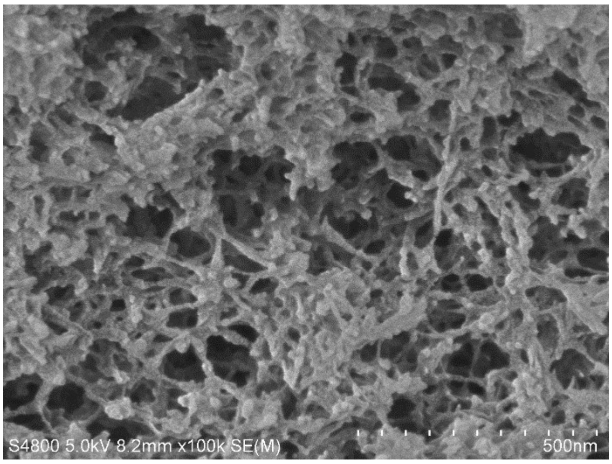

Figure S7. SEM image of JUC-522.

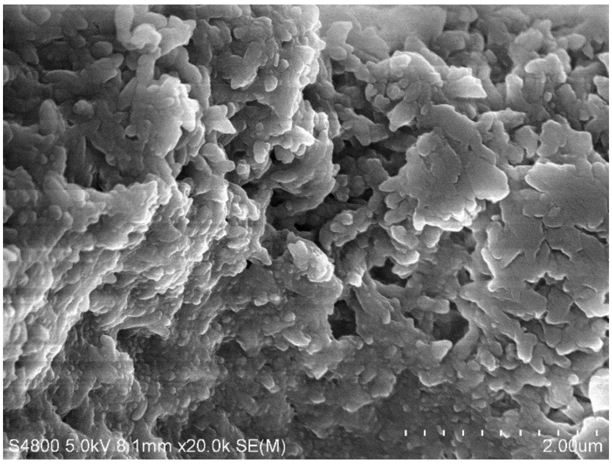

Figure S8. SEM image of JUC-523.

Section 4: FT-IR spectra

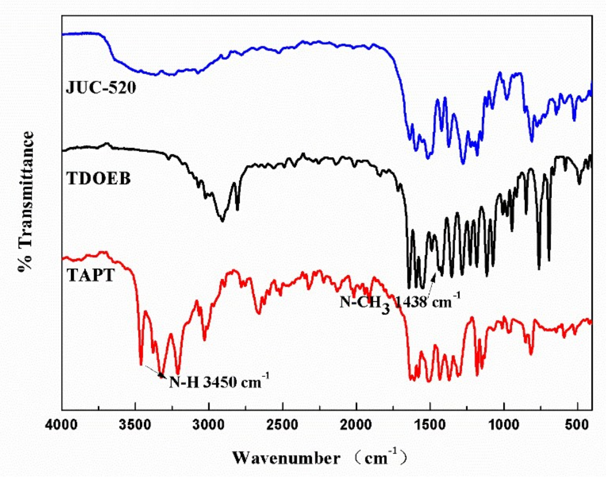

Figure S9. FT-IR spectra of JUC-520 (blue), TDOEB (black) and TAPT (red).

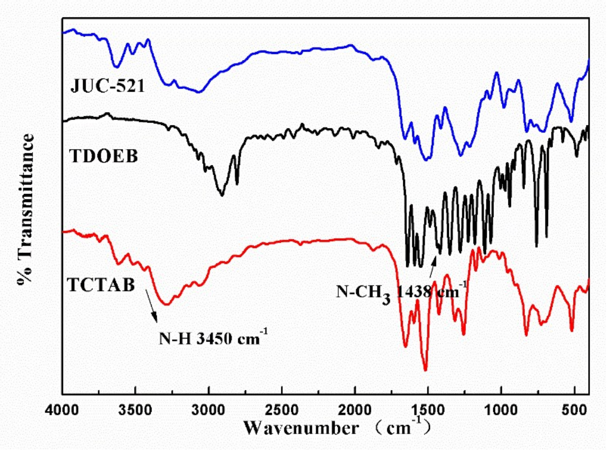

Figure S10. FT-IR spectra of JUC-521 (blue), TDOEB (black) and TCTAB (red).

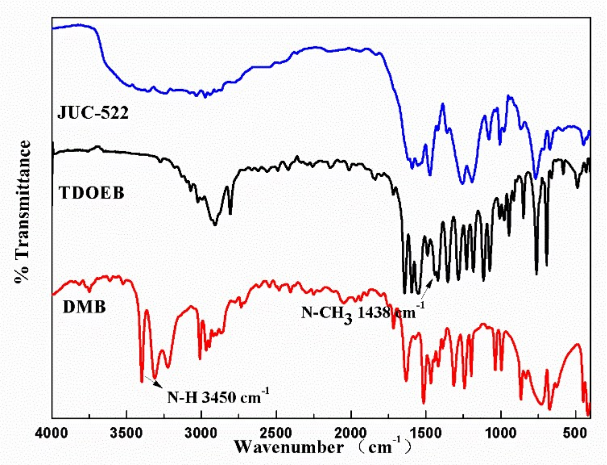

Figure S11. FT-IR spectra of JUC-522 (blue), TDOEB (black) and DMB (red).

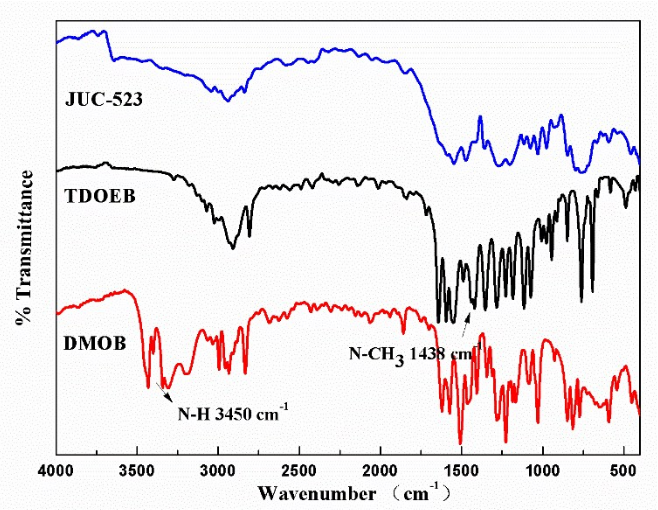

Figure S12. FT-IR spectra of JUC-523 (blue), TDOEB (black) and DMOB (red).

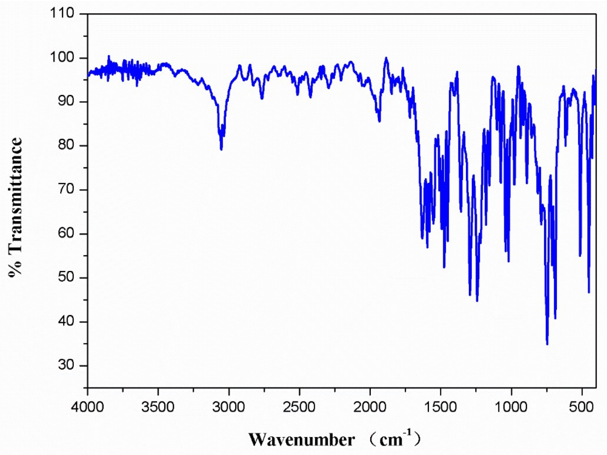

Figure S13. FT-IR spectrum of model compound, APPO.

Section 5: Solid-state ^{13}C NMR spectra

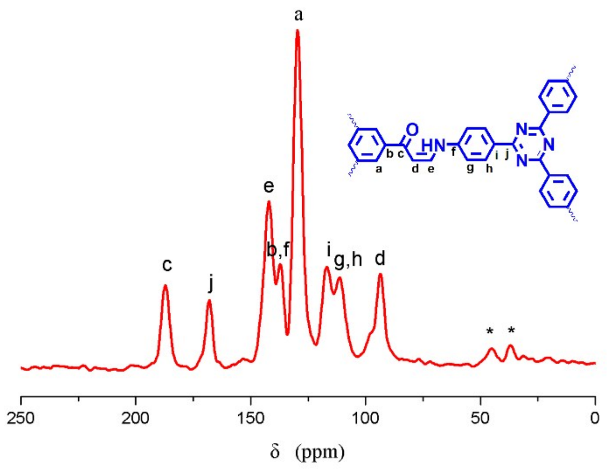

Figure S14. Solid-state ^{13}C NMR spectrum of JUC-520.

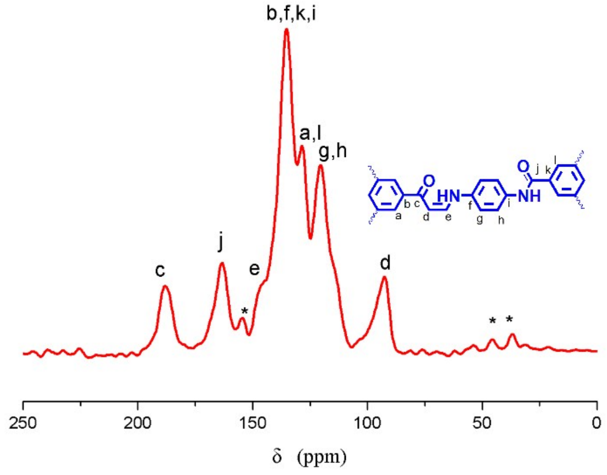

Figure S15. Solid-state ^{13}C NMR spectrum of JUC-521.

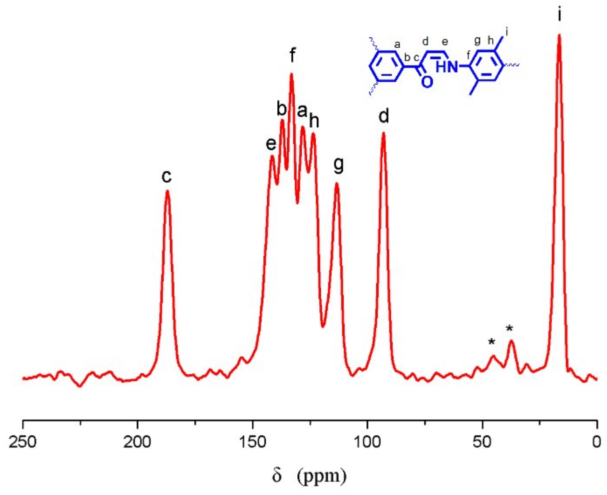

Figure S16. Solid-state ^{13}C NMR spectrum of JUC-522.

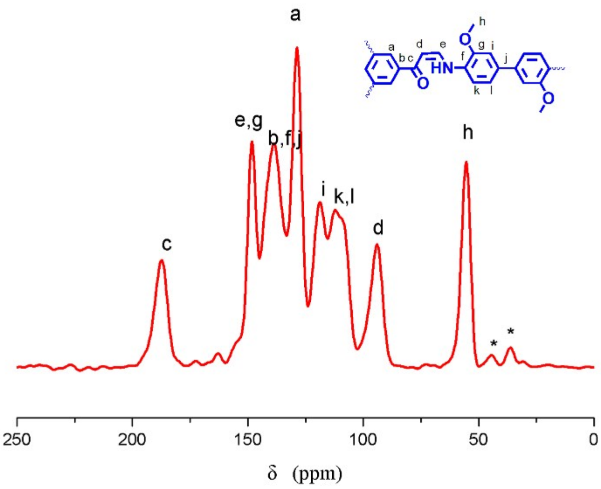

Figure S17. Solid-state ^{13}C NMR spectrum of JUC-523.

Section 6: TGA analysis

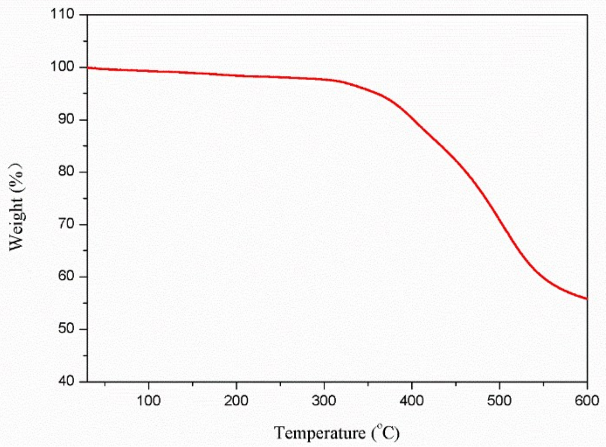

Figure S18. TGA curve of JUC-520.

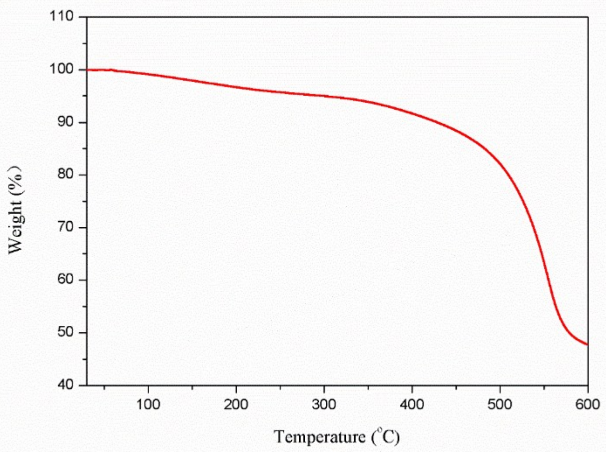

Figure S19. TGA curve of JUC-521.

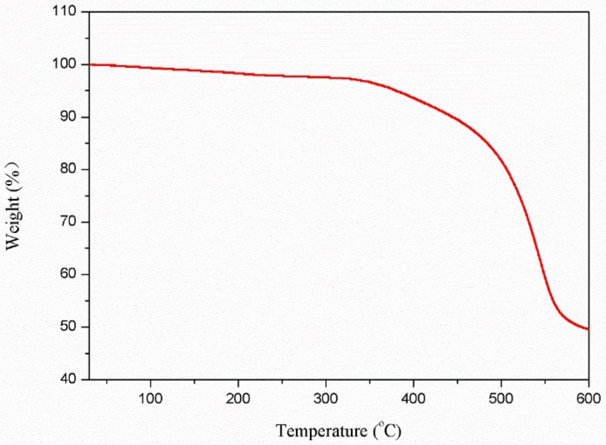

Figure S20. TGA curve of JUC-522.

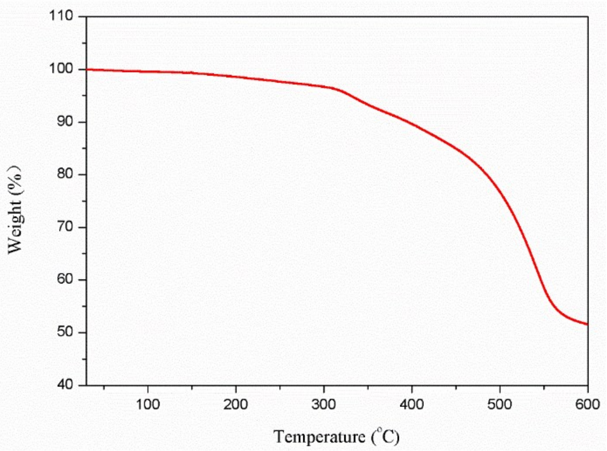

Figure S21. TGA curve of JUC-523.

Section 7: Stability test

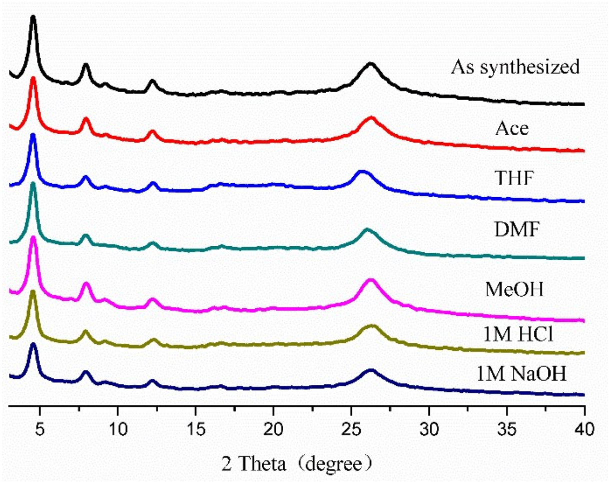

Figure S22. PXRD patterns of JUC-520 after the treatment in a variety of organic solvents and acid (1.0 M HCl) and base (1.0 M NaOH) aqueous solutions for 3 days.

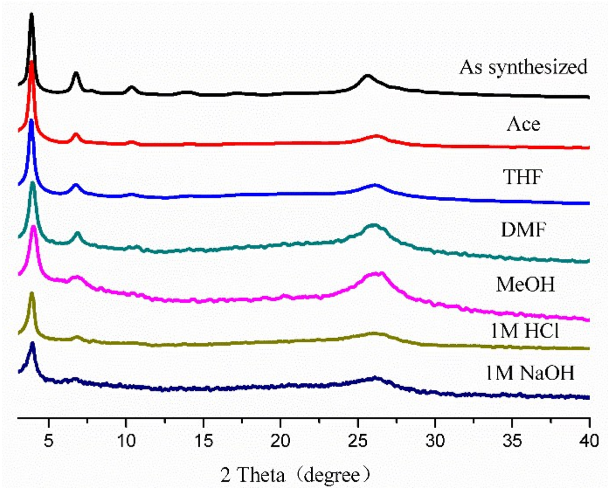

(a)

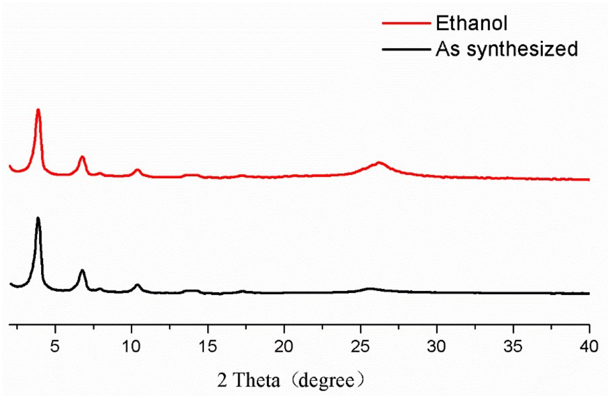

(b)

Figure S23. PXRD patterns of JUC-521 after the treatment in (a) a variety of organic solvents and acid (1.0 M HCl) and base (1.0 M NaOH) aqueous solutions and (b) in ethanol for 3 days.

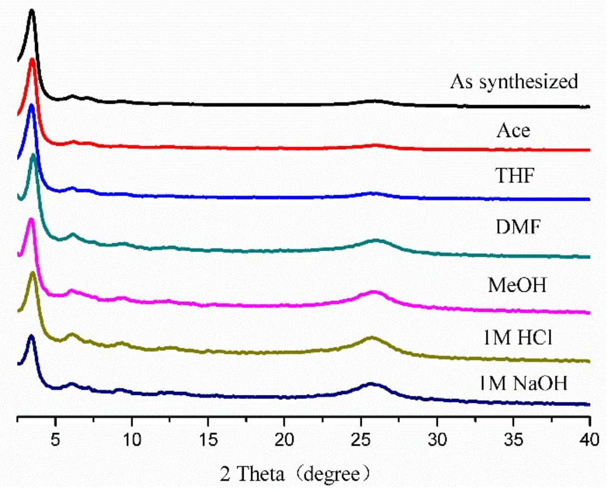

Figure S24. PXRD patterns of JUC-522 after the treatment in a variety of organic solvents and acid (1.0 M HCl) and base (1.0 M NaOH) aqueous solutions for 3 days.

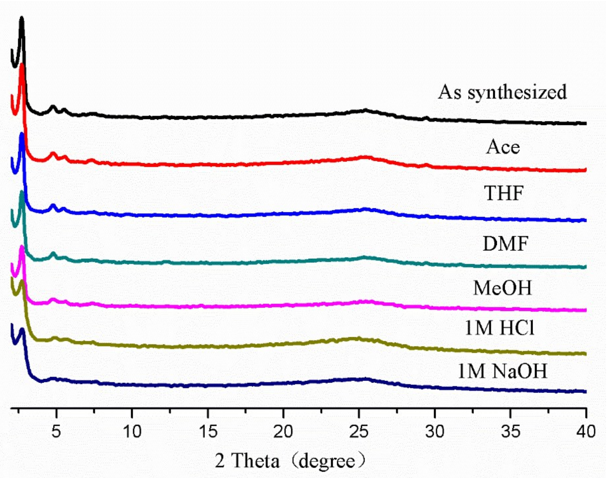

Figure S25. PXRD patterns of JUC-523 after the treatment in a variety of organic solvents and acid (1.0 M HCl) and base (1.0 M NaOH) aqueous solutions for 3 days.

Section 8: PXRD patterns and structures

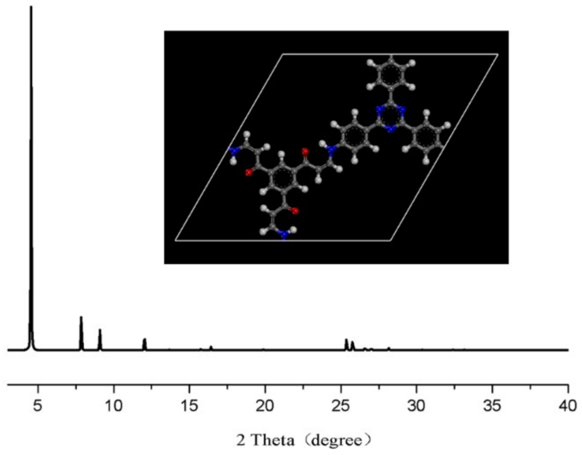

Figure S26. Calculated PXRD pattern of JUC-520 based on the eclipsed **bnn** net.

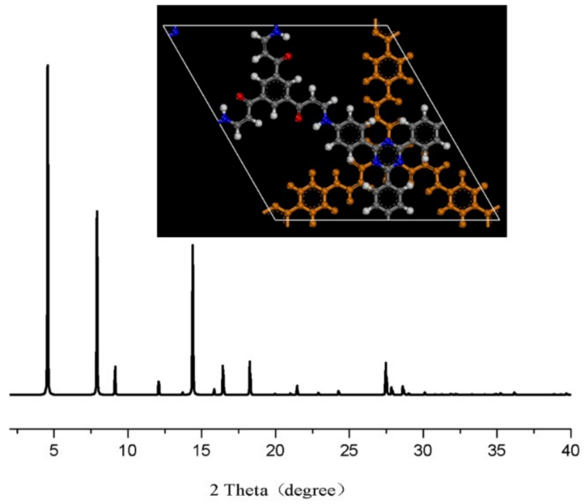

Figure S27. Calculated PXRD pattern of JUC-520 based on the staggered **bnn** net.

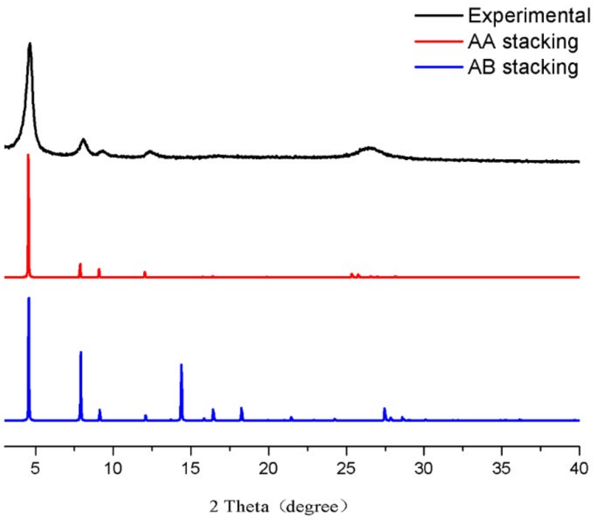

Figure S28. Comparison of PXRD patterns of JUC-520.

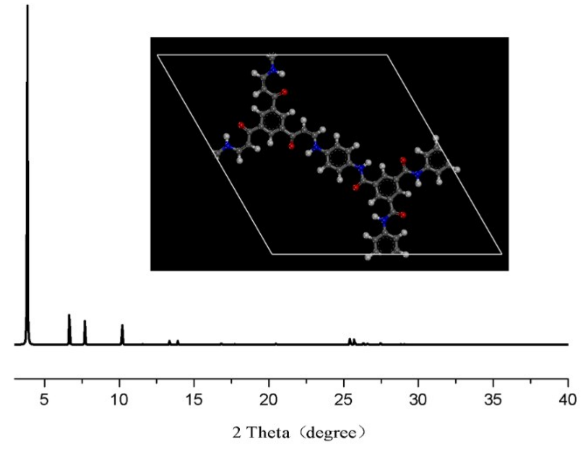

Figure S29. Calculated PXRD pattern of JUC-521 based on the eclipsed **bnn** net.

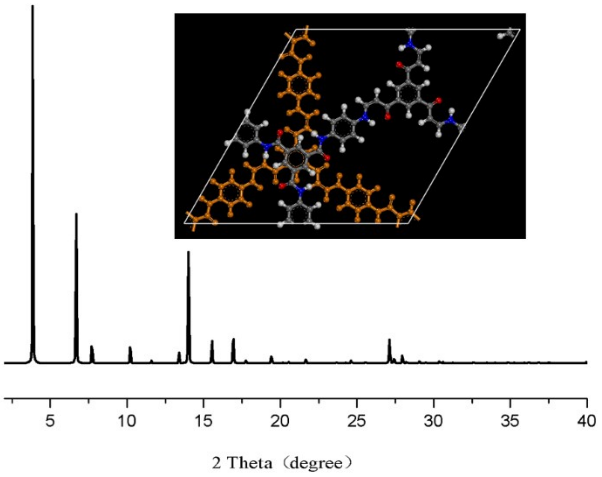

Figure S30. Calculated PXRD pattern of JUC-521 based on the staggered **bnn** net.

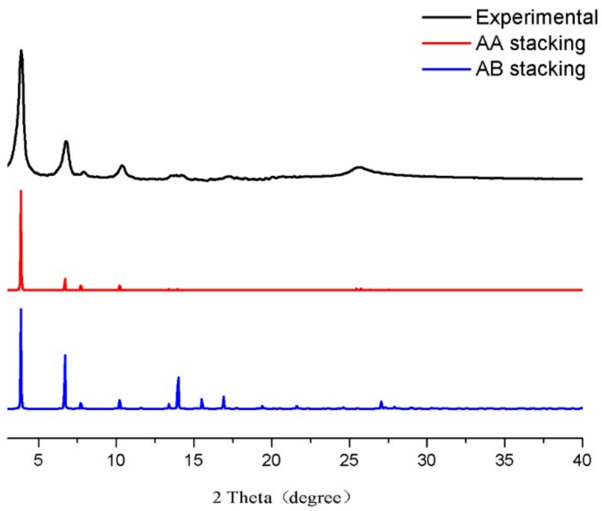

Figure S31. Comparison of PXRD patterns of JUC-521.

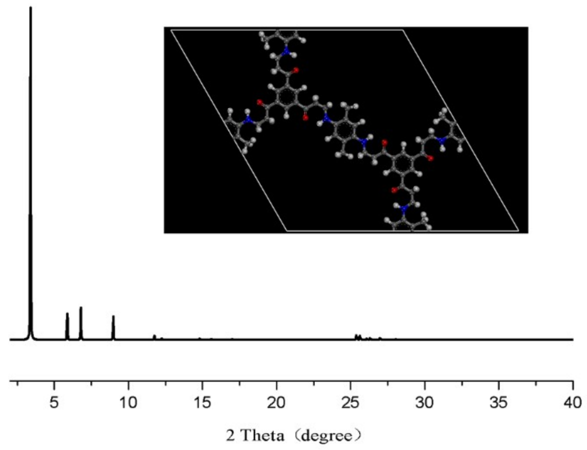

Figure S32. Calculated PXRD pattern of JUC-522 based on the eclipsed **bnn** net.

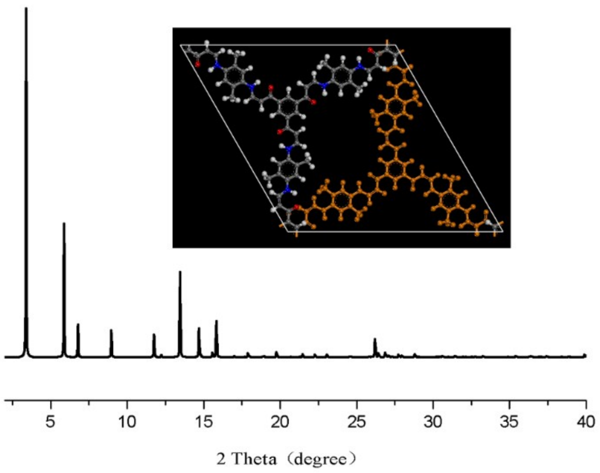

Figure S33. Calculated PXRD pattern of JUC-522 based on the staggered **bnn** net.

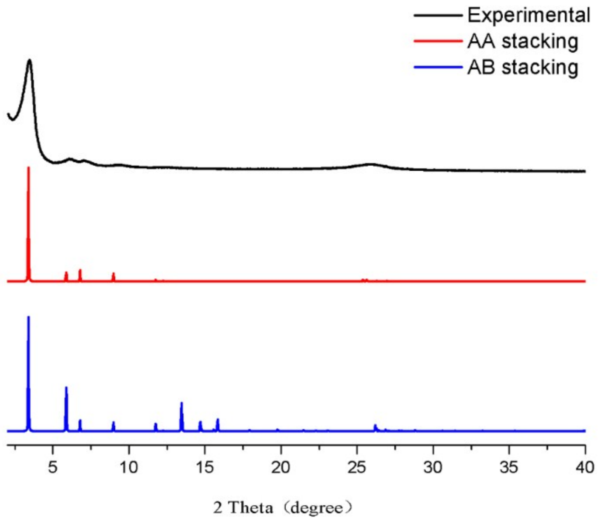

Figure S34. Comparison of PXRD patterns of JUC-522.

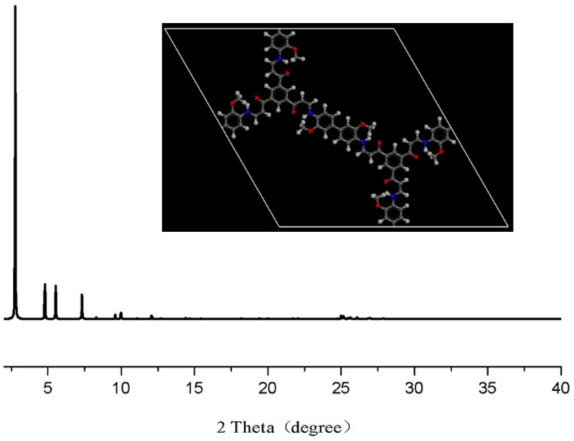

Figure S35. Calculated PXRD pattern of JUC-523 based on the eclipsed **bnn** net.

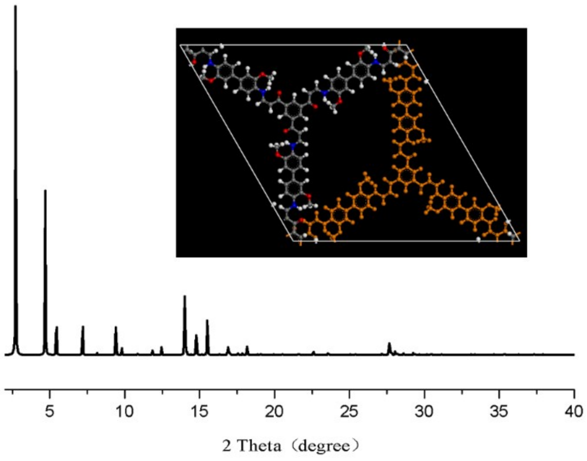

Figure S36. Calculated PXRD pattern of JUC-523 based on the staggered **bnn** net.

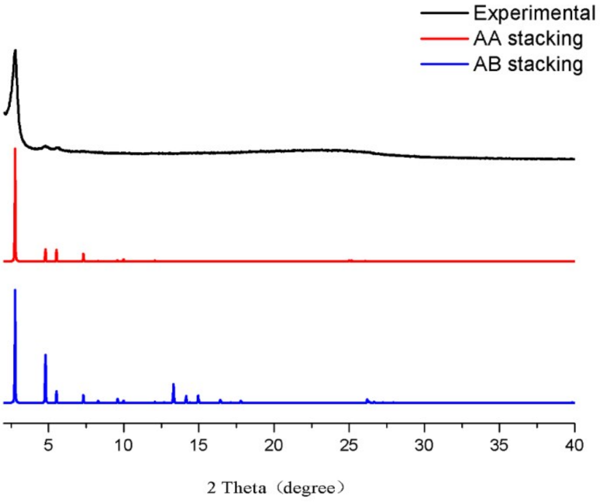

Figure S37. Comparison of PXRD patterns of JUC-523.

Section 9: Nitrogen adsorption

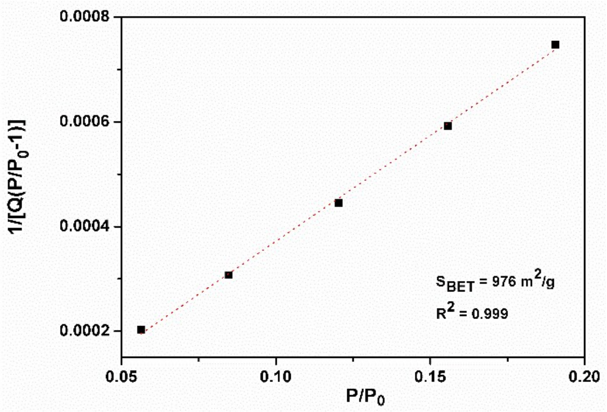

Figure S38. BET plot of JUC-520 calculated from N_2 adsorption isotherm at 77 K.

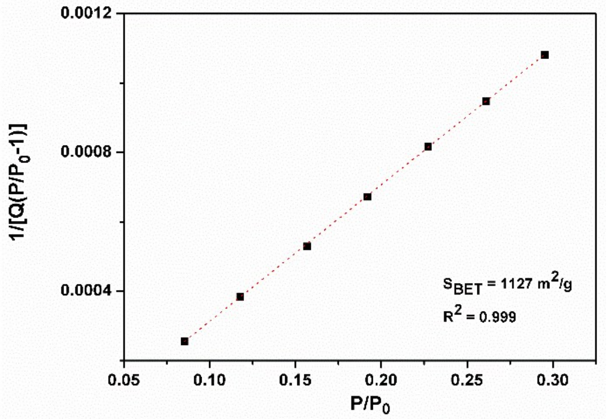

Figure S39. BET plot of JUC-521 calculated from N_2 adsorption isotherm at 77 K.

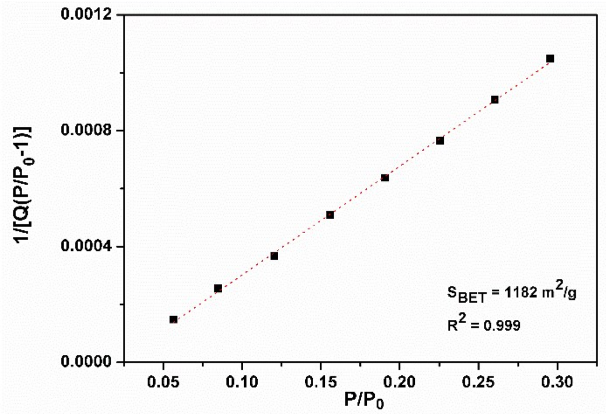

Figure S40. BET plot of JUC-522 calculated from N_2 adsorption isotherm at 77 K.

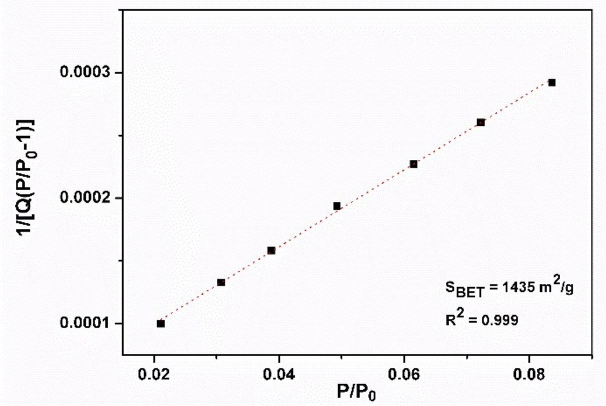

Figure S41. BET plot of JUC-523 calculated from N_2 adsorption isotherm at 77 K.

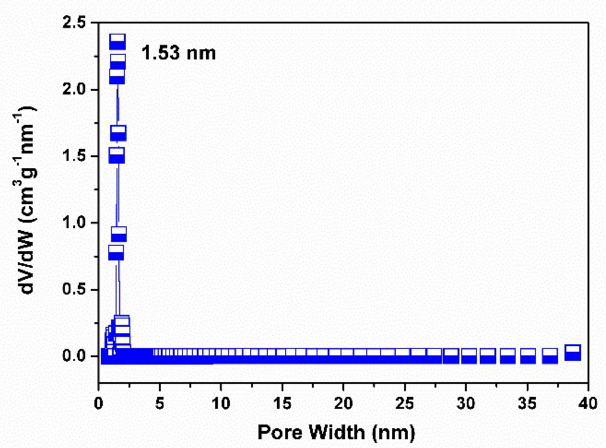

Figure S42. The pore-size distribution of JUC-520 estimated by nonlocal density functional theory (NLDFT).

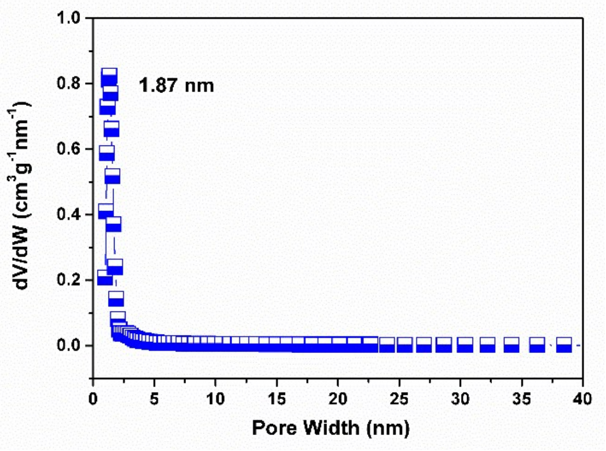

Figure S43. The pore-size distribution of JUC-521 estimated by NLDFT.

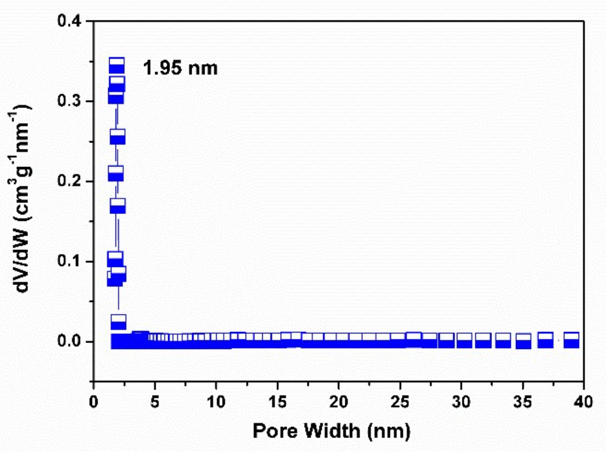

Figure S44. The pore-size distribution of JUC-522 estimated by NLDFT.

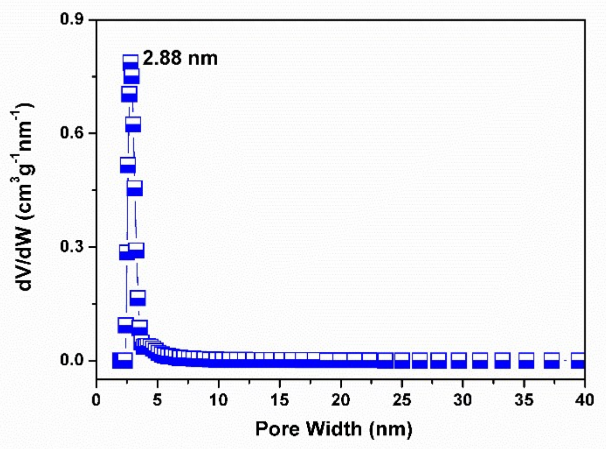

Figure S45. The pore-size distribution of JUC-523 estimated by NLDFT.

Section 10: Characterization of JUC-521-Fe

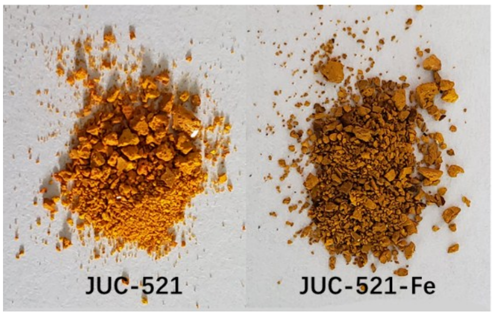

Figure S46. Comparison of digtital photographs of JUC-521 (left) and JUC-521-Fe (right).

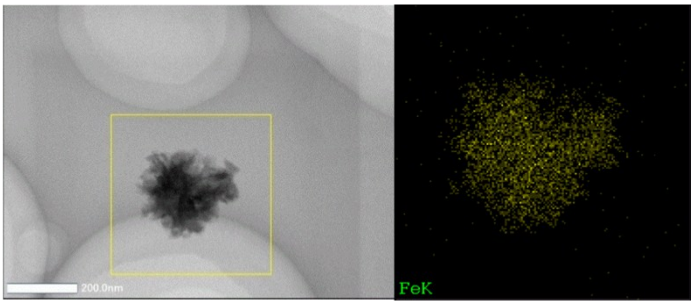

Figure S47. EDS mapping images of JUC-521-Fe.

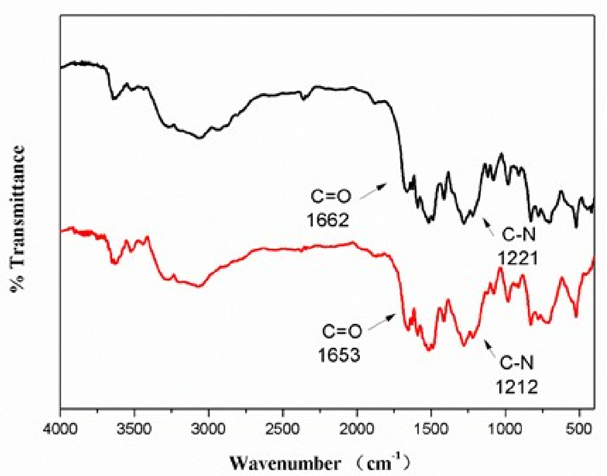

Figure S48. Comparison of FT-IR spectroscopy of JUC-521 (black) and JUC-521-Fe (red).

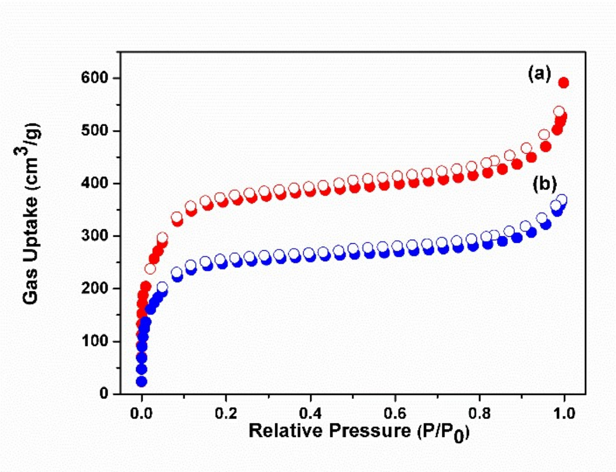

Figure S49. Comparison of N_2 adsorption of JUC-521 (a) and JUC-521-Fe (b).

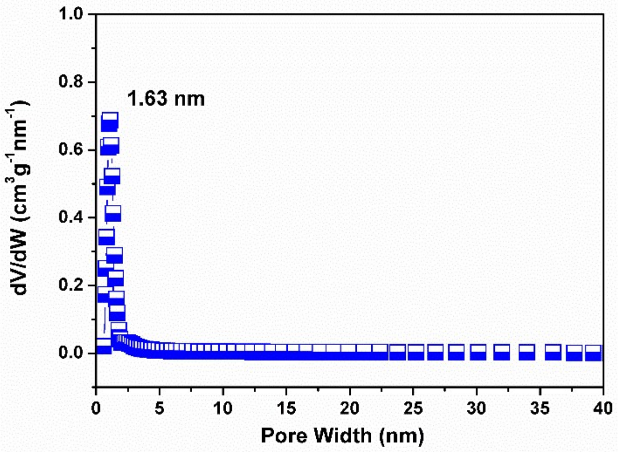

Figure S50. The pore-size distribution of JUC-521-Fe estimated by NLDFT.

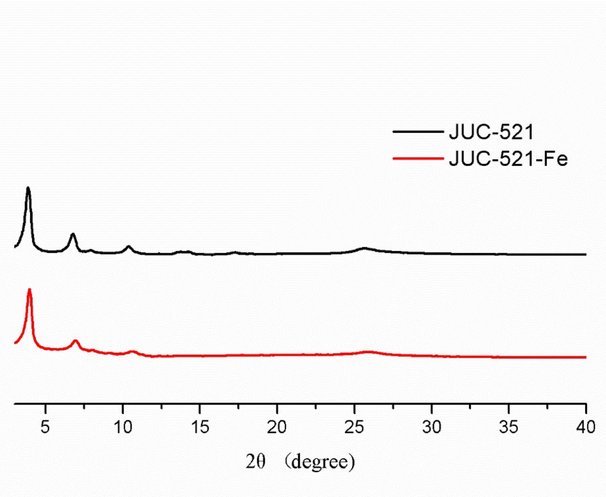

Figure S51. Comparison of PXRD patterns of JUC-521 (black) and JUC-521-Fe (red).

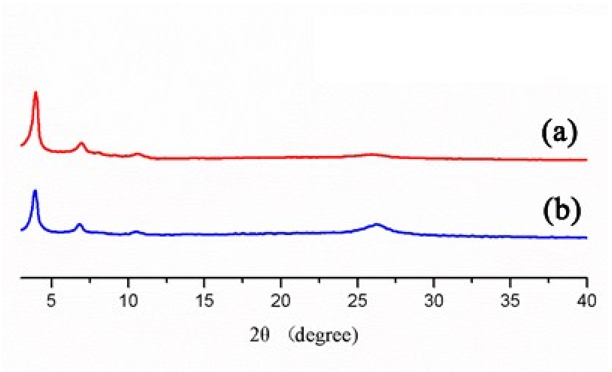

Figure S52. Comparison of PXRD patterns of JUC-521-Fe before (a, red) and after (b, blue) the Fenton reaction.

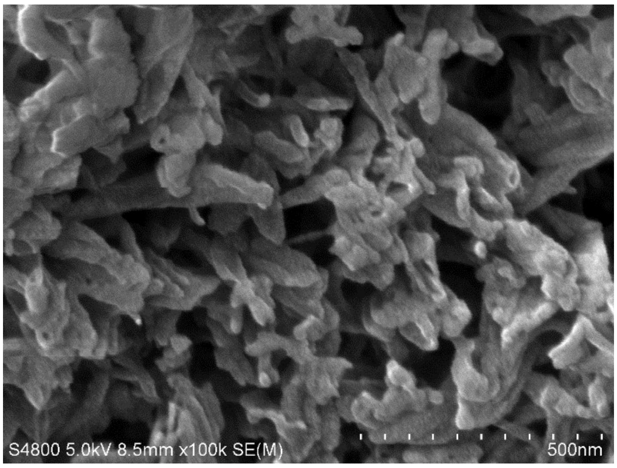

Figure S53. SEM image of JUC-521-Fe after the Fenton reaction.

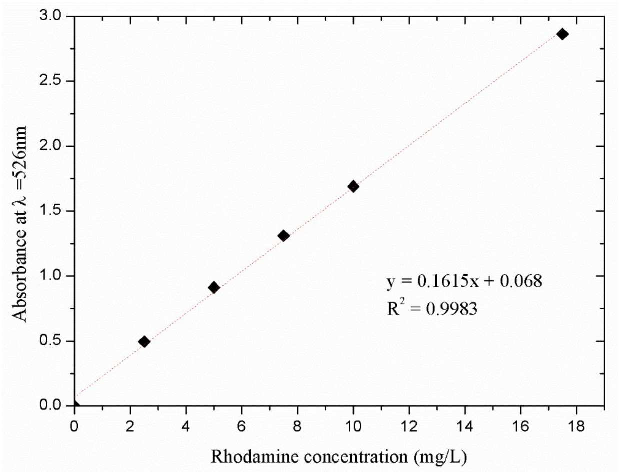

Figure S54. The standard UV-vis curve of Rh6G.

Section 11: Unit cell parameters and fractional atomic coordinates

Table S1. Unit cell parameters and fractional atomic coordinates for JUC-520 calculated on the basis of eclipsed **bnn** net.

Space group		*P*-6	
Calculated unit cell		$a = b = 22.4795$ Å, $c = 3.5114$ Å, $\alpha = \beta = 90°$ and $\gamma = 120°$	
Measured unit cell		$a = b = 22.7122$ Å, $c = 3.5302$ Å, $\alpha = \beta = 90°$ and $\gamma = 120°$	
Pawley refinement		ωRp = 3.63% and Rp = 2.52%	
atoms	x	y	z
C1	0.36558	0.62674	0.5
C2	0.29379	0.59517	0.5
C3	0.51116	0.58587	0.5
C4	0.47487	0.61807	0.5
O5	0.36378	0.52206	0.5
C6	0.62995	0.36905	0.5
N7	0.70197	0.40492	0.5
C8	0.39907	0.58456	0.5
C9	0.5914	0.40658	0.5
C10	0.62632	0.47869	0.5
C11	0.58994	0.51405	0.5
C12	0.51794	0.47775	0.5
C13	0.48288	0.40601	0.5
C14	0.51928	0.37066	0.5
N15	0.47953	0.5127	0.5
H16	0.26106	0.53675	0.5
H17	0.56968	0.61688	0.5
H18	0.50239	0.67659	0.5
H19	0.68487	0.50889	0.5
H20	0.6188	0.57261	0.5
H21	0.42433	0.37607	0.5
H22	0.48988	0.3121	0.5
H23	0.42269	0.48042	0.5

Table S2. Unit cell parameters and fractional atomic coordinates for JUC-520 calculated on the basis of staggered **bnn** net.

Space group		*P*-6	
Calculated unit cell		$a = b = 22.3738$ Å, $c = 6.4934$ Å, $\alpha = \beta = 90°$ and $\gamma = 120°$	
atoms	x	y	z
C1	0.26077	0.62671	0
C2	0.30103	0.5948	0
C3	0.07345	0.58604	0
C4	0.14229	0.6183	0
O5	0.15718	0.52167	0
C6	0.73694	0.36813	0
N7	0.70196	0.40324	0
C8	0.18467	0.58449	0
C9	0.81291	0.40574	0
C10	0.84984	0.47808	0
C11	0.92192	0.51373	0
C12	0.95792	0.47734	0
C13	0.92117	0.4052	0
C14	0.84909	0.36957	0
N15	0.03164	0.51253	0
H16	0.27508	0.53609	0
H17	0.0459	0.61728	0
H18	0.17353	0.6771	0
H19	0.82105	0.50812	0
H20	0.95166	0.57256	0
H21	0.95003	0.37523	0
H22	0.81967	0.31073	0
H23	0.05627	0.48005	0
C24	0.59415	0.29342	0.5
C25	0.63436	0.26149	0.5
C26	0.40713	0.25307	0.5
C27	0.47594	0.28525	0.5
O28	0.49063	0.18848	0.5
C29	0.07033	0.03486	0.5
N30	0.03527	0.06993	0.5

C31	0.51816	0.2513	0.5
C32	0.14644	0.07258	0.5
C33	0.18342	0.14496	0.5
C34	0.25554	0.18067	0.5
C35	0.29159	0.14433	0.5
C36	0.25487	0.0722	0.5
C37	0.18274	0.03651	0.5
N38	0.36531	0.17956	0.5
H39	0.60837	0.20278	0.5
H40	0.37963	0.28437	0.5
H41	0.50724	0.34404	0.5
H42	0.15465	0.17502	0.5
H43	0.28525	0.2395	0.5
H44	0.28374	0.04225	0.5
H45	0.15342	-0.02233	0.5
H46	0.38994	0.14708	0.5

Table S3. Unit cell parameters and fractional atomic coordinates for JUC-521 calculated on the basis of eclipsed **bnn** net.

Space group		P-6	
Calculated unit cell		$a = b = 26.4476$ Å, $c = 3.5064$ Å, $\alpha = \beta = 90°$ and $\gamma = 120°$	
Measured unit cell		$a = b = 26.5261$ Å, $c = 3.5154$ Å, $\alpha = \beta = 90°$ and $\gamma = 120°$	
Pawley refinement		ωRp = 5.34% and Rp = 3.92%	
atoms	x	y	z
C1	0.36292	0.6348	0.5
C2	0.30177	0.60579	0.5
C3	0.49102	0.60661	0.5
C4	0.45823	0.63191	0.5
O5	0.36549	0.54788	0.5
C6	0.62575	0.35251	0.5
C7	0.68568	0.39296	0.5
C8	0.39369	0.60113	0.5
C9	0.56774	0.46122	0.5
C10	0.59533	0.52217	0.5
C11	0.56233	0.55011	0.5
C12	0.50116	0.51717	0.5
C13	0.47349	0.45625	0.5
C14	0.50655	0.42838	0.5
N15	0.46641	0.54467	0.5
C16	0.583	0.37272	0.5
N17	0.60311	0.43419	0.5
O18	0.53089	0.33717	0.5
H19	0.27562	0.55604	0.5
H20	0.54066	0.63463	0.5
H21	0.47984	0.68154	0.5
H22	0.70153	0.44175	0.5
H23	0.64502	0.54938	0.5
H24	0.58512	0.59982	0.5
H25	0.42379	0.42912	0.5
H26	0.48351	0.37865	0.5
H27	0.41829	0.51567	0.5
H28	0.65143	0.46158	0.5

Table S4. Unit cell parameters and fractional atomic coordinates for JUC-521 calculated on the basis of staggered **bnn** net.

Space group		*P*-6	
Calculated unit cell		$a = b = 26.4383$ Å, $c = 6.5749$ Å, $\alpha = \beta = 90°$ and $\gamma = 120°$	
atoms	x	y	z
C1	0.36338	0.6352	0.5
C2	0.30218	0.60575	0.5
C3	0.49233	0.60807	0.5
C4	0.45924	0.63309	0.5
O5	0.36672	0.54865	0.5
C6	0.626	0.35281	0.5
C7	0.68598	0.39304	0.5
C8	0.39462	0.60193	0.5
C9	0.56894	0.46225	0.5
C10	0.59675	0.52326	0.5
C11	0.56388	0.55139	0.5
C12	0.50266	0.51857	0.5
C13	0.47482	0.45762	0.5
C14	0.50772	0.42958	0.5
N15	0.46794	0.54613	0.5
C16	0.58354	0.37335	0.5
N17	0.60405	0.43493	0.5
O18	0.53135	0.33801	0.5
H19	0.27636	0.55597	0.5
H20	0.54196	0.63633	0.5
H21	0.48061	0.68272	0.5
H22	0.70207	0.44189	0.5
H23	0.64647	0.55032	0.5
H24	0.58682	0.60113	0.5
H25	0.4251	0.4306	0.5
H26	0.48453	0.37982	0.5
H27	0.41983	0.51696	0.5
H28	0.65241	0.46212	0.5
C29	0.69685	0.30205	0
C30	0.63567	0.27243	0

C31	0.82601	0.27547	0
C32	0.79283	0.30038	0
O33	0.70045	0.21571	0
C34	0.95944	0.01969	0
C35	0.01951	0.05977	0
C36	0.72822	0.26899	0
C37	0.90241	0.1294	0
C38	0.93026	0.19042	0
C39	0.89747	0.21864	0
C40	0.83626	0.1859	0
C41	0.80835	0.12493	0
C42	0.84118	0.0968	0
N43	0.80163	0.21353	0
C44	0.91701	0.0404	0
N45	0.93751	0.102	0
O46	0.8648	0.00511	0
H47	0.60999	0.22265	0
H48	0.87563	0.30381	0
H49	0.81411	0.35	0
H50	0.03577	0.10865	0
H51	0.97999	0.21745	0
H52	0.92048	0.26838	0
H53	0.75862	0.09797	0
H54	0.81791	0.04705	0
H55	0.75351	0.18436	0
H56	0.98587	0.12916	0

Table S5. Unit cell parameters and fractional atomic coordinates for JUC-522 calculated on the basis of eclipsed **bnn** net.

Space group		*P*6	
Calculated unit cell		$a = b = 30.0949$ Å, $c = 3.5075$ Å, $\alpha = \beta = 90°$ and $\gamma = 120°$	
Measured unit cell		$a = b = 30.1108$ Å, $c = 3.5936$ Å, $\alpha = \beta = 90°$ and $\gamma = 120°$	
Pawley refinement		ωRp = 3.77% and *R*p = 2.45%	
atoms	x	y	z
C1	0.35892	0.63827	0.5
C2	0.30521	0.61318	0.5
C3	0.47067	0.61225	0.5
C4	0.44224	0.63489	0.5
O5	0.36042	0.5615	0.5
C6	0.38554	0.60827	0.5
C7	0.52396	0.46985	0.5
C8	0.55317	0.52336	0.5
C9	0.5302	0.55379	0.5
N10	0.44859	0.55778	0.5
C11	0.56505	0.61045	0.5
H12	0.28192	0.56947	0.5
H13	0.51432	0.63655	0.5
H14	0.46159	0.67854	0.5
H15	0.59685	0.5432	0.5
H16	0.40625	0.5326	0.5
H17	0.57316	0.62503	0.19366
H18	0.60263	0.62014	0.64682
H19	0.54603	0.62954	0.65952

Table S6. Unit cell parameters and fractional atomic coordinates for JUC-522 calculated on the basis of staggered **bnn** net.

Space group		*P*6_3	
Calculated unit cell		$a = b$ = 30.0988 Å, c = 6.5324 Å, $\alpha = \beta$ = 90° and γ = 120°	
atoms	x	y	z
C1	0.69221	0.30487	0.25
C2	0.63848	0.27984	0.25
C3	0.8039	0.27863	0.25
C4	0.77549	0.30132	0.25
O5	0.6936	0.22802	0.25
C6	0.71878	0.27479	0.25
C7	0.85699	0.136	0.25
C8	0.88627	0.18952	0.25
C9	0.86339	0.22003	0.25
N10	0.78182	0.22416	0.25
C11	0.97432	0.02829	0.25
C12	0.02802	0.05348	0.25
C13	0.86238	0.05387	0.25
C14	0.89092	0.03136	0.25
O15	0.97266	0.10493	0.25
C16	0.94762	0.05815	0.25
C17	0.80922	0.19646	0.25
C18	0.77995	0.14293	0.25
C19	0.80283	0.11242	0.25
N20	0.8844	0.10833	0.25
C21	0.89833	0.27668	0.25
C22	0.76793	0.05575	0.25
H23	0.61514	0.23614	0.25
H24	0.84755	0.30289	0.25
H25	0.79487	0.34496	0.25
H26	0.92994	0.20929	0.25
H27	0.73949	0.19899	0.25
H28	0.05064	0.0972	0.25
H29	0.81875	0.02948	0.25
H30	0.87168	0.98773	0.25

H31	0.73627	0.12315	0.25
H32	0.92699	0.12952	0.25
H33	0.90642	0.29127	0.08552
H34	0.9359	0.2863	0.32869
H35	0.87939	0.29583	0.3358
H36	0.7877	0.03649	0.32875
H37	0.75822	0.04143	0.08551
H38	0.73115	0.04597	0.33574

Table S7. Unit cell parameters and fractional atomic coordinates for JUC-523 calculated on the basis of eclipsed **bnn** net.

Space group		*P*6	
Calculated unit cell		$a = b = 36.9035$ Å, $c = 3.5605$ Å, $\alpha = \beta = 90°$ and $\gamma = 120°$	
Measured unit cell		$a = b = 36.8467$ Å, $c = 3.5395$ Å, $\alpha = \beta = 90°$ and $\gamma = 120°$	
Pawley refinement		ωRp = 3.91% and Rp = 2.55%	
atoms	x	y	z
C1	0.3528	0.64219	0.5
C2	0.30909	0.62313	0.5
C3	0.44064	0.61707	0.5
C4	0.41911	0.63655	0.5
O5	0.3514	0.57828	0.5
C6	0.373	0.61633	0.5
N7	0.42065	0.57311	0.5
O8	0.37779	0.48284	0.5
C9	0.35427	0.49947	0.5
C10	0.44295	0.55203	0.5
C11	0.42138	0.50822	0.5
C12	0.44439	0.48827	0.5
C13	0.48814	0.51085	0.5
C14	0.50904	0.55427	0.5
C15	0.4867	0.57459	0.5
H16	0.28902	0.58756	0.5
H17	0.4763	0.63579	0.5
H18	0.43604	0.6722	0.5
H19	0.32401	0.47821	0.64883
H20	0.34734	0.50437	0.20238
H21	0.37067	0.53054	0.64879
H22	0.42749	0.45262	0.5
H23	0.5447	0.57278	0.5
H24	0.50383	0.61025	0.5

Table S8. Unit cell parameters and fractional atomic coordinates for JUC-523 calculated on the basis of staggered **bnn** net.

Space group		$P6_3$	
Calculated unit cell		$a = b = 37.6289$ Å, $c = 6.4273$ Å, $\alpha = \beta = 90°$ and $\gamma = 120°$	
atoms	x	y	z
C1	0.68517	0.30875	0.25
C2	0.64233	0.29067	0.25
C3	0.7707	0.28212	0.25
C4	0.74958	0.30196	0.25
O5	0.68268	0.24553	0.25
C6	0.70438	0.28279	0.25
N7	0.75138	0.23833	0.25
O8	0.71105	0.14597	0.25
C9	0.68445	0.16255	0.25
C10	0.77387	0.21683	0.25
C11	0.75382	0.17309	0.25
C12	0.77796	0.15438	0.25
C13	0.82094	0.17723	0.25
C14	0.84016	0.22007	0.25
C15	0.81693	0.23953	0.25
C16	0.98163	0.02569	0.25
C17	0.02427	0.04377	0.25
C18	0.89582	0.05153	0.25
C19	0.91712	0.03191	0.25
O20	0.98383	0.08871	0.25
C21	0.96231	0.05143	0.25
N22	0.91506	0.09531	0.25
O23	0.95543	0.18763	0.25
C24	0.98204	0.17105	0.25
C25	0.89257	0.11679	0.25
C26	0.91265	0.16052	0.25
C27	0.88851	0.17924	0.25
C28	0.84554	0.15639	0.25
C29	0.8263	0.11355	0.25
C30	0.84952	0.09408	0.25

H31	0.62219	0.25583	0.25
H32	0.80567	0.30015	0.25
H33	0.76652	0.33694	0.25
H34	0.71739	0.21934	0.25
H35	0.6544	0.14021	0.33004
H36	0.67798	0.16774	0.08283
H37	0.69963	0.19302	0.33713
H38	0.76273	0.11949	0.25
H39	0.87508	0.23932	0.25
H40	0.83347	0.27449	0.25
H41	0.98163	0.02569	0.42737
H42	0.03632	0.06496	0.10912
H43	0.03401	0.06397	0.39482
H44	0.86085	0.03333	0.25
H45	0.90034	-0.00307	0.25
H46	0.94904	0.11435	0.25
H47	0.98981	0.16737	0.08283
H48	0.96623	0.13983	0.33001
H49	1.01145	0.19265	0.33716
H50	0.90375	0.21413	0.25
H51	0.79138	0.0943	0.25
H52	0.83299	0.05912	0.25

Section 12: References

(1) Pleier, A. K.; Glas, H.; Grosche, M.; Sirsch, P.; Thiel, W. R. Microwave assisted synthesis of 1-aryl-3-dimethylaminoprop-2-enones: a simple and rapid access to 3 (5)-arylpyrazoles. *Synthesis*, **2001**, *01*, 0055.

(2) Dos Santos, C. M. G.; Boyle, E. M.; De Solis, S.; Kruger, P. E.; Gunnlaugsson, T. Selective and tuneable recognition of anions using C_{3v}-symmetrical tripodal urea-amide receptor platforms. *Chem. Commun.*, **2011**, *47*, 12176.

(3) Mahmud, T.; Rehman, R.; Gulzar, A.; Khalid, A.; Anwar, J.; Shafique, U.; Salman, M. Synthesis, characterization and study of antibacterial activity of enaminone complexes of zinc and iron. *Arab. J. Chem.*, **2010**, *3*, 219.

(4) Board, W. J.; Wooten, R. G.; Whatley, C. W. Method for producing dimethylacetamide. *U. S. Patent*, No. 3342862, **1967**.
